# Chronotype differences in the risk of cancers, diabetes mellitus, and poor mental health among shift workers: a meta-analysis

**DOI:** 10.5271/sjweh.4271

**Published:** 2026-03-01

**Authors:** Beixi Li, Feng Wang, Natalie HY Tang, Anke Huss, Joey Wing-Yan Chan, Yun Kwok Wing, Lap Ah Tse

**Affiliations:** 1 JC School of Public Health and Primary Care, the Chinese University of Hong Kong, Hong Kong SAR, China.; 2 Shenzhen Municipal Key Laboratory for Health Risk Analysis, Shenzhen Research Institute of the Chinese University of Hong Kong, Shenzhen , China.; 3 Department Population Health Sciences, Utrecht University, Utrecht , The Netherlands.; 4 Department of Psychiatry, Faculty of Medicine, The Chinese University of Hong Kong, Hong Kong SAR, China.

**Keywords:** chronotype, diabetes, mental health, shift work

## Abstract

**Objective:**

Shift work is associated with various leading causes of premature death, which has been linked with individuals with specific chronotypes. This study synthesized evidence on chronotype’s role in associations between ever shift work and health outcomes.

**Methods:**

Six databases were searched (inception–September 2025) for cohort/case-control studies assessing chronotype-specific shift work impacts on breast/prostate cancer, diabetes mellitus, and mental health. PRISMA guidelines were used for reporting.

**Results:**

Fourteen studies were included in the review, comprising 2247 breast cancer cases, 3045 prostate cancer cases, 336 218 participants in diabetes studies, and 2128 poor mental health cases. Compared to daytime workers, both night shift workers with morning or evening chronotypes were more susceptible to breast cancer [morning type: pooled odds ratio (OR) 1.54, 95% confidence interval (CI) 1.01–2.37; evening type: pooled OR 1.41, 95% CI 1.04–1.90) and poor mental health (morning type: pooled OR 1.19, 95% CI 1.12–1.27; evening type: pooled OR 1.11, 95% CI 1.05–1.17]. Notably, night shift workers with evening chronotype were 84% more likely to develop prostate cancer than daytime workers. A positive dose–response relationship was identified between cumulative years of night shifts and prostate cancer among night shift workers with evening chronotype, indicating a 2.1% increase in risk for each additional year (P=0.012).

**Conclusions:**

Chronotype-matched scheduling does not effectively mitigate night shift risks. Nevertheless, evening chronotype night shift workers are particularly susceptible to various chronic non-communicable diseases, with a notable positive dose–response relationship observed between prostate cancer and evening chronotype night shift workers.

Individuals in modern industrialized societies have access to artificial light around the clock, which leads to varying preferences in their sleep-wake cycles, known as chronotypes ( 1). Early risers who are more active in the morning are considered to have a morning chronotype, while those who are more active at night are identified as an evening chronotype (ie, a state of eveningness) ( 2). Research suggests that the evening chronotype might be associated with greater risks of cancers, diabetes mellitus, and mental health disorders ( 3– 5). Potential mechanisms linking evening chronotype to these health issues include circadian misalignment, clock gene dysfunction, and prolonged exposure to artificial light at night ( 6, 7).

Notably, these same mechanisms are often experienced by shift workers, particularly those on night shifts. Due to work hours that conflict with the natural circadian rhythm, night shift workers frequently undergo circadian misalignment, sleep deprivation, and prolonged exposure to artificial light at night ( 8), making them more susceptible to same spectrum of health disorder ( 7, 9, 10), creating a state of forced eveningness. Despite the elevated health risks associated with night shift work and evening chronotype, research indicates that individuals with work schedules aligned to their chronotype tend to have higher job satisfaction and better work performance ( 11). In 2013, Juda et al ( 12) observed that night shift workers, a work schedule misaligned with an individual’s chronotype was associated with shortened sleep duration, greater social jetlag, and higher levels of sleep disturbances. Since then, research on the relationship between chronotype and various health impacts has expanded ( 5). However, comprehensive evidence regarding the effects of chronotypes and shift work schedules on the risks of cancers, diabetes mellitus, and poor mental health remains unclear ( 13). Therefore, this study aimed to synthesize existing evidence from observational studies on the association between shift workers with different chronotypes and multiple health outcomes, including breast cancer, prostate cancer, diabetes mellitus, and poor mental health and to critically evaluate the practical implications for chronotype-informed scheduling as a potential strategy to mitigate major health risks among shift workers.

## Methods

We followed the Preferred Reporting Items for Systematic Review and Meta-analysis (PRISMA 2020) ( 14) and Meta-analysis of Observational Studies in Epidemiology (MOOSE) reporting guidelines ( 15). This study was registered with PROSPERO.

### Search strategy

In this study, we focused on breast cancer and prostate cancer because the International Agency for Research on Cancer (IARC) Monographs highlight the causal link between shift work and the risk of these two cancer types ( 16). According to the World Health Organization, approximately 8% of the burden of depression can be attributed to workplace risk ( 17). The present study includes individuals with poor mental health, specifically those diagnosed with depression, anxiety, or mood disorders. The search included “mood disorders” to ensure comprehensive capture of studies on depressive conditions, which are classified under this umbrella term in medical indexing systems. We systematically searched six major databases: EMBASE, EBSCOhost (MEDLINE), APA PsycInfo, CINAHL, Web of Science, and PubMed. The search strategy involved using the title, abstract, and keywords, combining terms such as “shift work”, “chronotype”, “breast cancer”, “prostate cancer”, “diabetes”, and “mental health”. The publication range included studies from inception to February 2024, with regular updates until September 2025 (supplementary material, www.sjweh.fi/article/4271, supplement 1). Additionally, we manually searched for the references cited in the included articles. All literature retrieved was imported into Covidence, where duplicates were automatically removed. Two authors independently screened each record and reached a consensus on the final selection of eligible papers.

### Selection criteria

The inclusion criteria were: (i) observational studies with a cohort or case–control study design; (ii) chronotype measured using a validated questionnaire; (iii) health outcomes diagnosed by clinicians or assessed using validated diagnostic scales or self-reported diagnoses; (iv) sufficient statistical information available for combining effect sizes; and (v) shift workers identified as the subjects of interest. In cases of duplicated reports based on the same population, we selected the study with the largest sample size and the most detailed data, with clear definitions. Cross-sectional studies, reviews, commentaries, and interventional studies were excluded. Studies were excluded if the effect size of interest was not reported or could not be calculated. No language or geographical restrictions were applied.

### Data extraction

The following information was extracted from each eligible study: the first author, year of publication, country of origin, study design, sample size, gender distribution, work schedules (ie, daytime work, shift work, night shift work), chronotype, health outcomes (ie, breast cancer, prostate cancer, diabetes mellitus, poor mental health), and the corresponding risk estimates and their 95% confidence intervals (CI). Two authors independently extracted relevant data from each study in a Microsoft Excel datasheet.

### Risk of bias assessment

The Risk of Bias in Non-randomized Studies - of Exposures (ROBINS-E), which is specifically designed for evaluating observational studies, was employed to assess the quality of eligible studies ( 18). Two authors independently assessed the following seven domains of bias: (i) participants recruitment; (ii) exposure measurement; (iii) post-exposure interventions; (iv) control of confounding factors; (v) handling of missing data; (vi) outcome evaluation; and (vii) selective report of results. Each bias domain in ROBINS-E is evaluated using a series of signaling questions that aim to gather important information about the study and the analysis being assessed. After the relevant signaling questions have been completed, three considerations were made as follows: (i) the risk of bias with judgement of “low risk of bias”, “some concerns”, “high risk of bias”, and “very high risk of bias”; (ii) the predicted direction of bias; and (iii) whether the risk of bias (arising from this domain) is sufficiently high, in the context of its likely direction and the magnitude of the estimated exposure effect, to threaten conclusions about whether the exposure has an important effect on the outcome. An overall judgment was made for each of the three considerations. Judgments for the first and third considerations were derived from the domain-level judgments using an established algorithm. For bias domain-level judgments, justifications must be provided if the overall judgment suggested by the algorithm is overridden.

### Statistical analysis

Data analysis was conducted using the R program (version 4.2.0, R Foundation, Vienna, Austria) with the R package “meta” (8.1.0) and “dosresmeta” (v2.1.1). All data were transferred to odds ratio (OR) with a 95% CI before combination if only original counts were available. In cases where the effect size was reported as hazard ratio (HR) or relative risk (RR), these were treated as equivalent to the OR for consistency in the meta-analysis ( 19). A random-effects model was applied to combine OR and its corresponding 95% CI for each study. When studies reported separate effect sizes by subgroups, a combined effect size was calculated using a fixed-effect model before proceeding with subsequent analyses. The associations between different chronotypes, work schedules, and specific adverse health outcomes were examined separately. Publication bias was evaluated by Begg & Mazumdar’s rank test ( 20).

A random-effects dose–response meta-analysis was performed to estimate the relationship between cumulative indicators of night shifts and the risks of breast and prostate cancer, categorized by individual chronotypes. Subgroup analyses were conducted based on the associations between patterns of night shift work (ie, rotating versus permanent night shifts) and different chronotype. Leave-one-out approach was conducted to identify outlying or influential studies that drive heterogeneity. Subgroup analyses were conducted to investigate the heterogeneity sources from study designs (ie, cohort study and case-control study). For mental health studies, we excluded different categories of mental health issues (ie, depression and mood disorders).

## Results

### Study selection and characteristics

**Figure 1:**
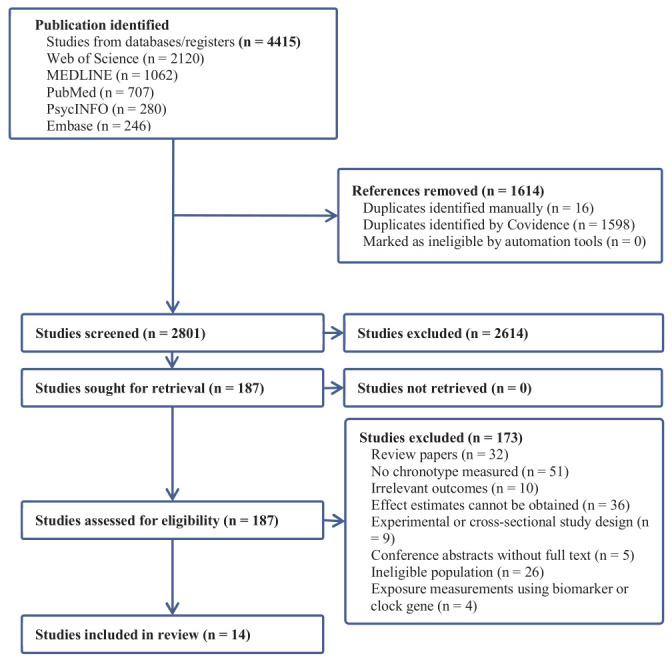
PRISMA flow diagram of the studies selection.

We identified a total of 4415 records from six databases. After removing 1614 duplicate records, 2801 records remained for title and abstract screening. Following this, we excluded 2614 ineligible records, resulting in 187 studies remaining for full assessment. Of them, 173 studies were further excluded due to reasons listed in figure 1, leaving 14 eligible records for further processing, including nine cohort studies ( 9, 10, 21– 27) and five case-control studies ( 28– 32) ( table 1). The ten cohort studies originated from six large cohorts, including the German Heinz Nixdorf Recall Cohort Study ( 22, 27), the Older Finnish Twin Cohort ( 21, 23), the UK Biobank ( 10, 26), The Netherlands Doetinchem Cohort Study ( 24), the Finnish Public Sector (FPS) study ( 9), and the United States Nurses’ Health Study II ( 25). The case–control studies were based on the Spanish Case–Control Study ( 30, 31), the Spanish CAPLIFE study ( 28), the French EPICAP Study ( 29), and the Danish Military Study ( 32).

**Table 1:** The association of adverse health outcomes between shift work schedule and chronotype. [MCTQ=Munich ChronoType Questionnaire; MEQ=Morningness–Eveningness Questionnaire; DTS=Diurnal Type Scale; CMR=cardiometabolic risk; PHQ-9=Patient Health Questionnaire; GHQ-12=General Health Questionnaire; Center for Epidemiologic Studies Depression Scale=CES-D; MT=morning type; ET=evening type; IT=intermediate type; NRNS=no rotating night shift work; RNS=rotating night shift work; OR _adj_=adjusted odds ratio; HR _adj_=adjusted hazard ratio; RR _adj_=adjusted relative risk; CI=confidence interval; n/e=not estimable; -=not reported].

Study ID Country, study design	Chronotype & Outcome assessment	Total sample / cases Chronotype (N) / Cases	Results: effect sizes
**Breast cancer**			
Schernhammer et al 2022 ( 21) Finland, cohort	Chronotype: DTS Outcome: Histology	5718/407 MT: - / 224 ET: - / 175 IT: - / 8	HR _adj_(95% CI), reference: day work of each type: MT: 2-shifts no nights: 0.91 (0.55–1.47); 3-shifts or nights only: 1.46 (0.93–2.28) ET: 2-shifts no nights: 0.73 (0.41, 1.31); 3-shifts or nights only: 1.56 (0.99–2.46)
Papantoniou et al 2016 ( 31) Spain, case–control	Chronotype: MCTQ Outcome: Medical record	3486/1708 MT: 1081/514 ET: 650/331 IT: 1123/536	OR _adj_(95% CI), reference: never night shift of each type MT: 1.17 (0.83–1.65) ET: 1.27 (0.81–2.00) IT: 1.17 (0.82–1.69)
Hansen et al 2012 ( 32) Denmark, case–control	Chronotype: Diurnal preference Outcome: Medical record	637/132 MT: 82 / 18 ET: 39 / 7 IT: 57 / 15	OR _adj_(95% CI), reference: never night shift of each type MT: < 884 nights 1.3 (0.5–3.7); ≥ 884 nights 3.9 (1.6–9.5) ET: < 884 nights 1.0 (0.3–4.0); ≥ 884 nights 0.7 (0.1–3.0) IT: < 884 nights 0.8 (0.2–3.0); ≥ 884 nights 2.0 (0.7– 5.8)
**Prostate cancer**			
Lozano-Lorca et al 2020 ( 28) Spain, case–control	Chronotype: MCTQ Outcome: Histology	875/465 MT: 530/283 ET: 79/45 IT: 255/130	OR _adj_(95% CI), reference: never night shift of each type MT: 1.25 (0.78–2.00) ET: 3.14 (0.91–10.76) IT: 1.71 (0.83–3.51)
Wendeu-Foyet et al 2018 ( 29) France, case–control	Chronotype: MEQ Outcome: Medical record	1693/818 MT: 598/301 ET: 255/113 IT: 839/403	OR _adj_(95% CI), reference: never night shift of each type MT: 0.77 (0.54–1.10) ET: 1.83 (1.05–3.19) IT: 0.96 (0.51–1.78)
Behrens et al 2017 ( 22) Germany, cohort	Chronotype: Mid-point of sleep Outcome: Medical record	1757/76 MT: 228/13 ET: 248/8 IT: 909/42	HR _adj_(95% CI), reference: 0–<1 year of shift of each type MT: 5.47 (1.45–20.71) ET: 1.20 (0.27–5.29) IT: 2.37 (1.26–4.45)
Dickerman et al 2016 ( 23) Finland, cohort	Chronotype: MEQ Outcome: Histology	11127/602 Definite MT: 3159/208 Somewhat MT: 3275/167 Somewhat ET: 3676/181 Definite ET: 1117/39	HR _adj_(95% CI), reference: Definite MT day shift Rotating shift: Definite MT: 1.0 (0.7–1.5) Somewhat MT: 0.5 (0.3–1.0) Definite ET: 1.5 (0.8–2.9) Somewhat ET: 1.5 (1.0–2.2)
Papantoniou et al 2014 ( 30) Spain, case–control	Chronotype: MCTQ Outcome: Medical record	2483/1095 MT: 993/452 ET: 270/125 IT: 720/307	OR _adj_(95% CI), reference: never night shift of each type MT: 1.14 (0.87–1.51) ET: 1.50 (0.85–2.66) IT: 1.02 (0.72–1.44)
**Diabetes mellitus**			
Hulsegge et al 2018 ( 24)Netherland, cohort	Chronotype: MEQ Outcome: Blood test	1061/- MT: 96 / - ET: 92 / - IT: 98 / -	OR _adj_(95% CI), reference: Never shift workers of each type T2D (≥ 11.1 mmol/l) for current shift workers: MT: 5.50 (0.86–35.11); ET: 1.20 (0.29–5.01); IT: 0.97 (0.11–8.18)
Vetter et al 2018 ( 10) United Kingdom, cohort	Chronotype: MEQ Outcome: Self-reported medical history and medication use	272214/6770 MT: 61131/1746 ET: 21879/676 IT: 15297/3576	OR _adj_(95% CI), reference: day worker of each type ^a^ MT SWP1: 1.15 (0.96–1.37); SWP2: 1.07 (0.85–1.33) SWP3: 1.14 (0.74–1.71); SWP4: 0.86 (0.59–1.22) ET SWP1: 1.11 (0.81–1.51); SWP2: 1.55 (1.08–2.19) SWP3: 1.53 (0.87–2.55); SWP4: 0.99 (0.67–1.42) IT SWP1: 1.14 (1.00–1.29); SWP2: 1.07 (0.91–1.26) SWP3: 1.40 (1.07–1.82); SWP4: 1.21 (0.95–1.51)
Vetter et al 2015 ( 25) United States, cohort	Chronotype: MEQ Outcome: Self-reported diagnosis	64615/319 MT: 22089/93 ET: 7029/49 IT: 33825/177	OR _adj_(95% CI), reference: IT MT NRNS: 0.75 (0.44–1.29); RNS: <10 yrs 0.91 (0.67–1.25); ≥10 yrs 1.63 (0.79–3.34) ET NRNS: 1.43 (0.77–2.62) ; RNS: <10 yrs 0.86 (0.57–1.32); ≥10 yrs 1.01 (0.43–2.37)
**Poor mental health**			
Liu et al 2023 ( 26) United Kingdom, cohort	Chronotype: MEQ Outcome: Clinical diagnosis (Depression)	220651/ 7902 MT: 136986/ 4650 ET: 83665/3252	HR _adi_(95% CI), reference: No shift work-MT No shift work- ET: 1.07 (1.02–1.13) Evening/weekend shifts- MT: 1.20 (1.10–1.32); ET: 1.11 (0.99–1.25) Irregular night shifts- MT: 1.18 (1.04–1.34); ET: 1.19 (1.03–1.37) Permanent night shifts- MT: 1.25 (1.08–1.46); ET: 1.04 (0.89–1.21)
Behrens et al 2021 ( 27)Germany, cohort	Chronotype: Mid-point of sleep Outcome: PHQ-9/ antidepressant medication (Depression)	Men: 295/19 Women: 91/28 Men: MT: 38 / 5; ET: 44 / 3: IT: 201 / 11 Women: MT: 34 / 9:ET: 28 / 6: IT: 117 / 13	RR _adj_(95% CI), reference: Never shift work/ <1 year of each type Men: MT: 0.50 (0.08, 2.98); ET: 17.4 (1.49, 203.3); IT: 0.67 (0.20, 2.28) Women: MT:1.83 (0.67, 4.98); ET: n/e; IT: 0.72 (0.10, 4.89)
Cheng et al 2021 ( 9) Finland, cohort	Chronotype: DTS Outcome: GHQ-12 (Mood disorders)	10637/2242 Definite MT: 800 / - Somewhat MT: 1207 / - Somewhat ET: 1427 / - Definite ET: 830 / -	OR _adj_(95% CI), reference: day work of each type ^b^ Definite MT SWP1: 0.98 (0.68, 1.41); SWP2: 0.95 (0.65, 1.38); SWP3: 1.56 (0.60, 4.07) Somewhat MT SWP1: 0.98 (0.72, 1.35); SWP2: 0.98 (0.72, 1.35); SWP3: 0.87 (0.39, 1.93) Somewhat ET SWP1: 1.35 (1.00, 1.83); SWP2: 1.11 (0.83, 1.47); SWP3: 1.91 (1.09, 3.34) Definite ET SWP1: 1.02 (0.67, 1.56); SWP2: 1.75 (1.18, 2.60); SWP3: 2.05 (1.06, 3.98)

^a^SWP1: Shift workers, but only rarely, if ever night shifts; SWP2: Irregular or rotating shifts with some night shifts; SWP3: Irregular or rotating shifts with usual night shifts; SWP4: Permanent night shifts. ^b^SWP1: Shift work without night shifts; SWP2: Shift work with night shifts; SWP3: Fixed night work

Participants were recruited from eight countries: Denmark ( 32), Finland ( 9, 21, 23), France ( 29), Germany ( 22, 27), Netherlands ( 24), Spain ( 30, 31), the United Kingdom ( 10, 26), and the United States ( 10, 25), Regarding adverse health consequences, three studies focusing on breast cancer included 9841 participants, reporting 2247 cases ( 21, 31, 32). Five studies investigating prostate cancer involved 17 891 workers, identifying 3045 cases ( 22, 23, 28– 30). Additionally, three cohort studies related to diabetes mellitus recruited a total of 336 218 workers ( 10, 24, 25). Finally, four studies on poor mental health involved 568 844 workers, reporting 2128 cases of poor mental health (supplementary table S1) ( 9, 26, 27). Except the Hulsegge study, which did not provide a case number for diabetes mellitus ( 24), a total of 15 425 adverse health events were reported (supplementary table S1).

The definitions of shift work and night shift work are detailed in supplementary table S2. In general, shift work is defined as work outside of 07:00–18:00 hours, while night shift work refers to work 00:00–05:00 or 06:00 hours. Shift workers had experience ranging from 1–>30 years. The average age of participants varied from 40 to >70 years, with both males and females recruited for studies on diabetes mellitus and poor mental health. For the purposes of meta-analysis, shift work exposure was standardized into a hierarchical structure. The broadest category, ever shift work, includes all shift types. The more specific category, ever night shift work, includes only individuals whose work schedule specifically involves working during the night period. Furthermore, for cancer outcomes, we conducted subgroup analyses comparing rotating night shift work ( 21, 28– 31) to permanent night shift work ( 28– 31), as a sufficient number of studies provided directly comparable definitions for these specific exposures. For diabetes and mental health outcomes, definitions were often not disaggregated beyond ever night shift or were too different to pool quantitatively; for instance, studies used incompatible classification systems based on night shift type (eg, permanent/rotating) ( 24, 25, 27) versus frequency of night shifts (rarely, if ever night shifts/some night shifts/usual night shifts/permanent night shifts) ( 9, 10, 26).

Chronotypes was assessed using various methods, including the mid-point of sleep ( 22, 27), a single question from the Diurnal Type Scale (DTS) ( 9, 21, 32), the Munich ChronoType Questionnaire (MCTQ) ( 28, 30, 31), the Morningness-Eveningness Questionnaire (MEQ) or a validated question derived from MEQ ( table 1) ( 10, 23– 26, 29). We employed a standardized approach to synthesize these measurements into three consistent categories: morning type (MT), intermediate type (IT), and evening type (ET). The most common assessment method was a single question derived from MEQ or DTS ( 9, 10, 21, 23– 26, 32), which was employed in nine studies. For these studies, “definite morning” responses were categorized as MT, “definite evening” as ET, and “somewhat morning” or “somewhat evening” responses were classified as IT. The MCTQ was used in three studies, where established cut-points were based on the calculated mid-sleep time on free days: values earlier than 04:00 hours were categorized as MT, 04:01–05:00 hours as IT, and later than 05:00 hours as ET ( 28, 30, 31). Two studies determined chronotype based on the calculated mid-point of sleep, using the following categorization: sleep midpoints earlier than 03:00 hours were classified as MT, 03:00–04:00 hours as IT, and later than 04:00 hours as ET ( 22, 27). One study using the full MEQ questionnaire maintained its original morning, intermediate, and evening classification provided by the original authors ( 29).

Cancer outcomes were determined through histology or medical records, while diabetes mellitus was assessed by blood tests, physical examinations, medical records, or self-reported diagnoses and medications. Poor mental health was evaluated using a validated mental health scale, self-reports of prescribed antidepressant medications, or physician/clinician diagnoses ( table 1).

### Risk of bias

Overall, the included studies had low to medium risk of bias. As shown in supplementary figure S1, according to the seven risks of bias domains, six studies were considered to have a low risk of bias ( 21, 22, 27– 31). The rest of the studies had a medium risk of bias ( 9, 23– 26, 30– 32). The Cheng et al study ( 9) had a high risk of bias because they failed to control some key confounding variables that could lead to overestimate exposure, including the health condition of the participants and the family history of the related adverse health outcomes. Those who had some concerns of bias due to confounding factors were mainly failed to adjust the occupational factors which may not affect the effects estimation of the exposure ( 10, 23, 24, 32, 33). The high risk of bias arising from measurement of the exposure were found in one study as shift work was not clearly defined ( 23). A third high risk of bias pertained to the measurement of the outcome, where two studies relied on self-reported methods for determining the health outcome ( 10, 25). Moreover, three studies used a case–control study design which did not select controls from the community and did not assess the exposures from the reliable records or structured interview ( 30– 32). Those studies that have some concerns of bias mostly have their predicted direction of bias towards the null ( 10, 24, 25, 33), except for two studies with their predicted direction of bias toward harm of higher exposure ( 23, 32). The rest of the studies had a low risk of bias which would be most likely to turn the predicted direction of bias towards the null ( 22, 27– 31).

### Meta-analysis and sub-group analysis

As presented in table 2(with detailed forest plots in supplementary figure S2–3), shift workers with evening chronotype showed elevated risks across all evaluated health outcomes (prostate cancer, pooled OR: 1.67, 95% CI 1.21–2.29; poor mental health, pooled OR: 1.11, 95% CI 1.05–1.17) compared to daytime workers, although the risk of breast cancer was not significantly increased (pooled OR: 1.21, 95% CI 0.86–1.70) and diabetes (pooled OR: 1.17, 95% CI 0.98–1.41). We observed that shift workers with evening chronotype exhibited the highest risks for both prostate cancer and diabetes mellitus compared to daytime workers, followed by intermediate chronotype and morning chronotype shift workers. Conversely, shift workers with morning chronotype had the highest risks for breast cancer and poor mental health compared to daytime workers. Notably, shift workers with morning chronotype showed a significantly increased risk of poor mental health (pooled OR: 1.19, 95% CI 1.12–1.27). For intermediate chronotype shift workers, we observed a 15% increased risk of diabetes (pooled OR: 1.15, 95% CI 1.06–1.26) compared to daytime workers.

**Table 2:** Meta-analysis for the association between shift work, individual chronotype and the risk of cancers, diabetes, and poor mental health (OR, 95%CI). All pooled results presented in the tables are based on the fully adjusted effect estimates extracted from the original studies. [OR=odds ratio; CI=confidence intervals]. **BOLD signifies P<0.05.**

		Breast cancer			Prostate cancer			Diabetes			Poor mental health	
		OR	95% CI		OR	95% CI		OR	95% CI		OR	95% CI
Ever shift work ^a^												
	Morning type	1.36	0.94–1.96		1.06	0.85–1.33		1.06	0.95–1.19		**1.19**	**1.12–1.27**
	Intermediate type	1.13	0.81–1.59		1.19	0.79–1.79		**1.15**	**1.06–1.26**		1.12	0.97–1.30
	Evening type	1.21	0.86–1.70		**1.67**	**1.21–2.29**		1.17	0.98–1.41		1.11	1.05–1.17
Ever night shift work ^b^												
	Morning type	**1.54**	**1.01–2.37**		1.22	0.90–1.65		1.00	0.87–1.15		**1.20**	**1.12–1.28**
	Intermediate type	1.13	0.81–1.59		**1.47**	**1.06–2.04**		**1.19**	**1.02–1.38 ^c^**		1.12	0.93–1.35
	Evening type	**1.41**	**1.04–1.90**		**1.84**	**1.30–2.61**		1.19	0.94–1.52		**1.11**	**1.04–1.17**

^a^For ever shift work, the comparisons were daytime work or never shift work. ^b^For ever night shift work, the comparisons were daytime work or never night shift work. ^c^The association is based on one study data entry (Vetter et al. 2018).

Also shown in table 2, night shift workers exhibited further increased risk of disease outcomes compared to daytime workers, following similar patterns as shift workers with different chronotypes. Specifically, night shift workers with either morning or evening chronotypes demonstrated statistically significant increases in the risk of breast cancer (morning chronotype, pooled OR: 1.54, 95% CI 1.01–2.37; evening chronotype, pooled OR: 1.41, 95% CI 1.04–1.90) and poor mental health (morning chronotype, pooled OR=1.20, 95% CI 1.12–1.28; evening chronotype, pooled OR=1.11, 95%CI 1.04–1.17) comparing to daytime workers. Furthermore, while night shift workers with intermediate chronotype showed a 19% increased risk of diabetes mellitus (pooled OR: 1.19, 95% CI 1.02–1.38), night shift workers with evening chronotype exhibited an insignificant increase in the risk of diabetes mellitus (pooled OR: 1.19, 95% CI 0.94–1.52) compared to daytime workers.

We further grouped the patterns by night shift work in table 3(with detailed forest plots in supplementary figures S4–5), rotating night shift workers with evening chronotype had a 72% higher risk of prostate cancer (pooled OR: 1.72, 95% CI 1.03–2.89), while permanent night shift workers with evening chronotype had a 76% higher risk (pooled OR: 1.76, 95% CI 1.13–2.75) comparing to daytime workers. However, no significant association was observed for rotating and permanent night shift workers with different chronotypes and breast cancer.

**Table 3:** Meta-analysis for the association between rotating and permanent night shift work by chronotype and cancer. All pooled results presented in the tables are based on the fully adjusted effect estimates extracted from the original studies. [OR=odds ratio; CI=confidence intervals]. **BOLD signifies P<0.05.**

		Breast cancer			Prostate cancer	
		OR	95% CI		OR	95% CI
Rotating night work						
	Morning type	1.15	0.88–1.51		0.90	0.44-1.84
	Intermediate type	1.13	0.72–1.78 ^a^		1.12	0.83-1.51
	Evening type	1.20	0.75–1.91		**1.72**	**1.03-2.89**
Permanent night work						
	Morning type	1.26	0.76–2.09 ^a^		1.09	0.84–1.41
	Intermediate type	1.24	0.70–2.19 ^a^		0.96	0.74–1.25
	Evening type	1.11	**0.59–2.10 ^a^**		1.76	**1.13–2.75**

^a^One study data entry for permanent night shift work with breast cancer (Papantoniou et al. 2016).

**Figure 2:**
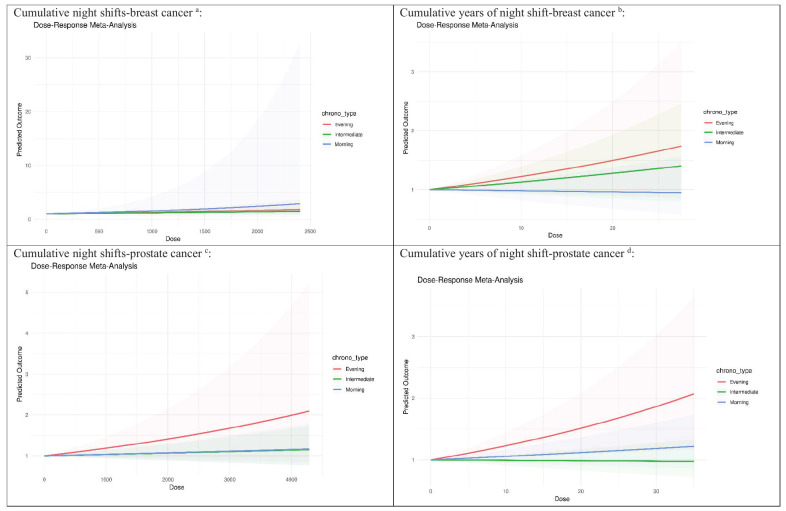
Exposure–response relationship between different chronotype night shift workers and breast or prostate cancer. ^a^Studies included (breast cancer): • Papantoniou-2016, cumulative nights of shifts: Never night work; 36–599 nights; 600–1799 nights; >=1800 nights. • Hansen-2012, cumulative nights of shifts: Never night work; <884 nights; >=884 nights. ^b^Studies included (breast cancer): Papantoniou-2016, cumulative years of night shift category: Never night work; 1-4 years; 5-14 years; >=15 years. ^c^Studies included (prostate cancer): • Wendeu-Foyet-2018, cumulative nights of shifts: Never night work; <1314 nights; ≥1314 nights. • Papantoniou-2014, cumulative nights of shifts: Never night work; <= 1152 nights; 1153–2856 nights; >=2857 nights. ^d^Studies included (prostate cancer): • Lozano-Lorca-2020, cumulative years of night shift category: Never night work; ≤7 years; 7–≤26 years; >26 years. • Wendeu-Foyet-2018, cumulative years of night shift category: Never night work; <10 years; 10-19 years; 20-29 years; ≥30 years. • Behrens-2017, cumulative years of night shift category: 0-<1 year; 1-<10 years; ≥10 years.

Figure 2 illustrated the exposure dose–response relationship between cumulative nights and cumulative years of night shifts in relation to the risks of breast and prostate cancer, categorized by chronotypes. While no significant trend was observed among night shift workers with breast cancer by different chronotypes, a positive gradient association between cumulative years of night shifts and prostate cancer with evening chronotype was observed, showing a 2.1% increase in risk for each additional year (P=0.012) (supplementary table S3).

### Sensitivity analysis and publication bias

Sensitivity analyses (supplementary figure S6) which combined studies with the same study designs (ie, case-control design) for breast cancer studies and prostate cancer studies, revealed a similar trend of risks as before; however, no statistically significant risk was observed for breast cancer. In the case of poor mental health studies, after removing the Cheng et al study (supplementary figure S6, c), which focused on mood disorders while the other studies investigated depression, the pooled risks remained similar to those before its removal and continued to be significant. There was no evidence showing publication bias from rank correlation test of funnel plot asymmetry (supplementary figure S7–8).

## Discussion

This study provides novel insights into how chronotype modulates the association between shift work and adverse health outcomes. Our findings demonstrate the risk patterns across chronotype groups, with evening chronotype night shift workers showing elevated risks for breast cancer, prostate cancer, and poor mental health. Notably, we observed a dose–response relationship between cumulative years of night shift work and prostate cancer risk specifically among evening chronotype workers.

Our findings must be interpreted within the context of the existing extensive literature on shift work and adverse health consequences. Lots of meta-analyses have established a significant association between night shift work and increased risk of breast cancer ( 34), prostate cancer ( 35, 36), diabetes mellites ( 37), and poor mental health ( 38, 39). The summary risk estimates from these studies (eg, reported OR typically of 1.06–1.43) represent an average effect across all shift workers. Our overall pooled estimates, which do not account for chronotype, are consistent with these previous findings (OR 1.13–1.23), lending credibility to our methodological approach.

However, the novel contribution of our study lies in revealing the significant heterogeneity hidden within this average risk. By stratifying by chronotype, we demonstrate that the health risk is not uniformly distributed across all shift workers. For instance, while the average risk for prostate cancer may be modest ( 22, 23, 28– 30), we found that it is concentrated almost exclusively among evening chronotypes (OR=1.84). Conversely, the average risk for diabetes masks a significant vulnerability among IT in our cohort ( 10, 24, 25). Notably, the established mental health risk appears to be primarily driven by MT, a finding previously obscured ( 9, 26, 27).

Therefore, our results do not contradict previous meta-analyses but rather provide a crucial explanatory layer. They suggest that chronotype maybe an effect modifier that can reconcile inconsistencies across previous studies and help identify the subgroups of workers who are most susceptible to the detrimental effects of shift work. This refines the prevailing understanding of shift work risk from a uniform hazard to a more complicated association between occupational exposure and individual circadian biology.

Despite extensive research on the association between shift work and adverse health outcomes, the role of chronotypes in this relationship is not frequently reported. Our meta-analysis reveals a complex pattern wherein the risk associated with shift work is differentially modified by chronotype, depending on the specific health outcome. While some studies suggest evening chronotype individuals may better tolerate night shifts due to reported longer sleep duration and higher sleep quality ( 11, 12), our findings reveal that significant health risks persist across chronotypes, challenging the assumption that subjective adaptability translates to long-term health protection. This heterogeneity suggests that the interaction between endogenous circadian physiology and exogenous shift work demands may involve distinct pathways for different diseases.

The observed health risks likely stem from multiple interconnected pathways. Night shift work and extreme chronotypes induce circadian misalignment, primarily through inappropriate light at night exposure. This disrupts the central circadian pacemaker and suppresses pineal melatonin secretion, a key regulator of circadian rhythms ( 36). Melatonin suppression and sleep disruption can subsequently dysregulate downstream hormonal pathways, including the levels of estrogen and testosterone, which are known to influence the growth of hormone-sensitive cancers ( 7, 40, 41). The particularly strong association between evening chronotype and cancer risk may be explained by a synergistic effect between genetic predisposition and environmental exposure (similar to the Knudson two-hit hypothesis, which is a foundational genetic model) ( 40). Specifically, polymorphisms in core circadian clock genes like *PER3*[associated with evening preference ( 42) and altered cancer risk ( 43, 44)] may confer a genetic vulnerability. When this predisposition is coupled with the environmental stressor of night shift work, which causes melatonin suppression and circadian disruption, the combination may have a multiplicative effect on risk ( 45). This gene-environment interaction provides a plausible biological mechanism for the dose-response relationship we observed between night shift duration and cancer risk in evening chronotype workers.

Notably, while the analysis of ever-exposure suggested a significantly increased breast cancer risk among evening chronotypes who had worked night shifts (OR=1.41, 95% CI 1.04–1.90), the dose–response analysis for cumulative exposure in this subgroup did not show a significant linear trend. This may suggest that for ET, the initial adoption of night shift work, which represents a significant misalignment with their natural circadian preference, is a primary driver of risk, rather than a straightforward cumulative dose-effect. Furthermore, this non-significant trend should be interpreted with caution, as the dose–response analysis for chronotype subgroups was limited to only two studies. The lack of a statistically significant association may be due to a lack of statistical power to precisely estimate a linear trend within these strata.

Our finding of elevated diabetes risk specifically among intermediate chronotype shift workers aligns with established mechanisms whereby circadian misalignment induces metabolic disturbances ( 5, 8, 46). As demonstrated in experimental models, light at night directly disrupts central circadian pacemakers and promotes metabolic syndrome ( 5, 8). This disruption is propagated to peripheral clocks in metabolic tissues, including the liver, pancreas, and adipose tissue, leading to a cascade of impairments that result in impaired insulin sensitivity and disrupted glucose metabolism ( 8). Intermediate chronotype workers present an intriguing case for this mechanism, where the significant elevated diabetes risk was most consistently observed in this type (ever shift work OR=1.15; ever night shift work OR=1.19). While their less pronounced circadian preferences might suggest better shift work tolerance, our results indicate this apparent flexibility may come at a metabolic cost.

The lack of a strong endogenous circadian signal in IT may result in a system that is more vulnerable to disintegration under shift work conditions, making their metabolic systems may be more susceptible to the disorganized effects of erratic light-dark cycles and irregular sleep patterns ( 8, 47). However, it is crucial to note that the association between night shift work and IT on diabetes is primarily based on a single, large cohort study ( 10), with limited data from one other investigation ( 25). This important limitation necessitates caution in interpreting the findings and highlights the need for replication in future independent studies. If validated, the chronic internal desynchronization experienced by IT could provide a compelling physiological explanation for this potential metabolic risk.

The association between shift work and poor mental health is most pronounced among morning chronotypes. This finding challenges the common assumption that ET would be most adversely affected by non-standard schedules. It suggests that the mismatch between innate circadian preference and environmental demands is a critical driver of mental health risk ( 5). For MT, whose biological rhythms are optimized for early rising and evening rest, compulsory night work represents a profound form of circadian misalignment. This misalignment is likely exacerbated by physiological hypersensitivity to phase-shifting cues. As Petrowski et al ( 41) demonstrated, MT exhibit a more reactive hypothalamic-pituitary-adrenal (HPA) axis, characterized by an elevated cortisol awakening response ( 41). The stress of working during their biological night may therefore dysregulate stress hormones to a greater degree in MT, potentiating depressive and anxiety disorders ( 41, 48). Conversely, ET may experience a relative attenuation of this stress response despite the misalignment, which could explain their more modest, yet still significant, increase in risk. Thus, the very biological traits that define the morning chronotype, a strong phase-advance and stress reactivity, may become vulnerability factors when placed in conflict with shift schedules.

The findings from our meta-analysis are consistent with the hypothesis that night shift work-induced circadian misalignment is a primary biological mechanism underlying these adverse health outcomes. This interpretation is supported by two key factors, first is the extensive experimental evidence demonstrating that circadian disruption directly promotes carcinogenesis and metabolic dysfunction ( 8, 16), and second is the fact that the individual studies included in our meta-analysis extensively adjusted for a wide range of potential confounding factors.

While chronotype modifies the magnitude of risk, the persistence of significant health risks across all chronotype groups suggests that chronotype-matched scheduling is unlikely to be a sufficient standalone strategy for mitigating major disease endpoints ( 11, 12). This finding underscores that the fundamental challenge remains the circadian disruption inherent to night work itself. Future interventions should therefore prioritize reducing this core disruption (eg, through optimized shift rotations and light management) alongside any consideration of individual chronotype ( 49). Nevertheless, we must acknowledge the heightened vulnerability of male night shift workers with evening chronotypes.

Our study has several strengths. While previous reviews have primarily focused on the association between night shift work and adverse health outcomes, none have examined the relationship between specific traits of shift workers and leading causes of premature death. We included both shift work exposure and chronotype in this review, providing a lifelong career perspective on the additive effects of night shift work and evening chronotype on cancer risk. However, several limitations should be acknowledged. First, the small number of studies available for each specific health outcome, particularly for the dose-response analyses, precludes strong conclusions and necessitates cautious interpretation. Nevertheless, the large sample sizes of the included cohorts provide robust initial insights that establish a valuable foundation for future research. Second, heterogeneity in the definitions of shift work and night shift work across the included studies represents a potential source of bias. Although we addressed this through a tiered classification system (ie, “ever shift work” versus “ever night shift work”), misclassification likely remains. Relatedly, the assessment of chronotype was not consistent across all studies, utilizing various questionnaires and cut-off points, which may have introduced measurement error despite our efforts to standardize categories. The lack of precise, payroll-based data on the number of specific shift types (morning, evening, night) and unmeasured variance likely stems from fundamental differences in shift schedules between North American and European studies, which could not be accounted for in our analysis. Third, although the primary studies adjusted for many major confounders, the possibility of residual or unmeasured confounding (eg, specific job-related stressors) cannot be ruled out, a limitation inherent to meta-analyses of observational data. Fourth, potential healthy worker effects may lead to an underestimation of true risks, as workers who cannot tolerate shift work may leave these jobs early in their careers. It is important to note that the net effect of these limitations, particularly non-differential misclassification, residual confounding, and the healthy worker effect, would most likely bias the observed effect estimates toward the null, meaning our results may underestimate the true risk of shift work and night shift work for the various diseases studied. Finally, we pragmatically pooled HR, RR, and OR to allow for a comprehensive meta-analysis. Although this is a common approach when outcomes are rare, we acknowledge that this assumes a degree of equivalence between these measures that may not be perfect and could introduce bias, however minimal. Future research should address these limitations through larger, prospective studies with chronotype-stratified designs, more standardized and detailed exposure assessments, and the collection of data on a wider range of potential confounding variables.

### Concluding remarks

In conclusion, our meta-analysis suggests that chronotypes modifies the association between shift work and the risk of several major health outcomes. A positive exposure-response relationship between prostate cancer and cumulative years of night work appears to be primarily restricted to evening chronotype shift workers. However, the current findings do not support the effectiveness of chronotype-matched shift scheduling as an optimal managerial strategy to mitigate the negative health effects of night shift work and the limited number of studies for each specific health outcome precludes definitive conclusions for single diseases. Future studies should prioritize larger, prospective studies with more precise exposure assessments, including detailed shift pattern data and potential genetic and actigraphy measures, to clarify these complex relationships.

## Supplementary material

Supplementary material

## Data Availability

The data collection forms, and other materials are publicly available. The study protocol and statistical analysis plan are available upon request to the corresponding author. Supplementary data will be available online.
