# Supplementary material for: Chronotype differences in the risk of cancers, diabetes mellitus, and poor mental health among shift workers: a meta-analysis
Source: Scand J Work Environ Health. 2026 Feb 28;52(2):98–109. doi: 10.5271/sjweh.4271 (PMC12955724; doi:10.5271/sjweh.4271)

Chronotype differences in the risk of cancers, diabetes mellitus, and poor mental health among shift workers: a meta-analysis[1]

by Beixi Li, PhD, Feng Wang, PhD, Natalie HY Tang, MPH, Anke Huss, PhD, Joey Wing-Yan Chan, FHKAM (Psychiatry), Yun Kwok Wing, FHKAM (Psychiatry), Lap Ah Tse, PhD[2]

1. Supplementary materials
2. Correspondence to: Prof. Lap Ah Tse, JC School of Public Health and Primary Care, the Chinese University of Hong Kong. 4/F School of Public Health and Primary Care, Prince of Wales Hospital, Sha Tin, N.T., Hong Kong SAR, 999077, China. [E-mail: shelly@cuhk.edu.hk]

Supplement 1 **Searching strategy**

Embase

S1 shift work.mp. or shift work/ or night shift.mp. or night work/ or night shift/ or nighttime work.mp.
S2 chronotype.mp. or diurnal preference.mp. or morningness.mp. or eveningness.mp. or morning type.mp. or evening type.mp.
S3 depression/ or depression.mp. or anxiety/ or anxiety.mp. or mood disorders.mp. or mood disorder/or breast cancer.mp. or breast cancer/ or prostate cancer.mp. or prostate cancer/ or diabetes.mp. or diabetes mellitus.mp.
S1 and S2 and S3

EBSCOhost (MEDLINE)

S1 shift work or night work or night shift or nighttime work
S2 chronotype or morningness or eveningness or diurnal preference or morning type or evening type
S3 depression OR anxiety OR mood disorders OR breast cancer OR prostate cancer OR diabetes
S4 S1 AND S2 AND S3

CINAHL

S1 shift work or night work or night shift or nighttime work
S2 chronotype or morningness or eveningness or diurnal preference or morning type or evening type
S3 depression OR anxiety OR mood disorders OR breast cancer OR prostate cancer diabetes
S4 S1 AND S2 AND S3

APA PsycInfo

S1 shift work.mp. or shift work/ or night shift.mp. or night work/ or night shift/ or nighttime work.mp.
S2 chronotype.mp. or diurnal preference.mp. or morningness.mp. or eveningness.mp. or morning type.mp. or evening type.mp.
S3 depression/ or depression.mp. or anxiety/ or anxiety.mp. or mood disorders.mp. or mood disorder/ or breast cancer.mp. or breast cancer/ or prostate cancer.mp. or prostate cancer/ or diabetes.mp. or diabetes mellitus
S1 and S2 and S3

Pubmed

("shift work"[Title/Abstract] OR "night work"[Title/Abstract] OR "nighttime work"[Title/Abstract] OR "night shift"[Title/Abstract] AND ("chronotype"[Title/Abstract] OR "diurnal preference "[Title/Abstract] OR "morningness"[Title/Abstract] OR "eveningness"[Title/Abstract] OR "morning type"[Title/Abstract] OR "evening type"[Title/Abstract] AND ("depression"[Title/Abstract] OR "anxiety"[Title/Abstract] OR "mood disorders"[Title/Abstract] OR "mood disorder"[Title/Abstract] OR "diabetes"[Title/Abstract] OR " diabetes mellitus "[Title/Abstract] OR "breast cancer"[Title/Abstract] OR "prostate cancer"[Title/Abstract])

Web of science

(TI=(shift work or night work or night shifts)) OR AB=(shift work or night work or night shifts) AND
(TI=(chronotype or morningness or eveningness or morning type or evening type)) OR AB=(chronotype or morningness or eveningness or morning type or evening type) AND
(TI=(depression or anxiety or mood disorder or mood disorders or diabetes or diabetes mellitus or breast cancer or prostate cancer)) OR AB=(depression or anxiety or mood disorder or mood disorders or diabetes or diabetes mellitus or breast cancer or prostate cancer

Last searched: 10 September 2025

Supplementary Table S1 **Sample size, the number of cases and controls in the included studies**

Author	Year	Disease	Original work patterns	**No. workers**	**No. cases**
Schernhammer	2022	breast cancer	day only	4,640	325
Schernhammer	2022	breast cancer	2-shifts without nights	614	33
Schernhammer	2022	breast cancer	3-shifts or nights only	464	49
Papantoniou	2016	breast cancer	Never night work	2,980	1,438
Papantoniou	2016	breast cancer	Ever night work	506	270
Hansen	2012	breast cancer	Never night work	450	89
Hansen	2012	breast cancer	Ever night work	187	43
**Sum**				**9,841**	**2,247**
Lozano-Lorca	2020	prostate cancer	Never night work	712	368
Lozano-Lorca	2020	prostate cancer	Ever night work	163	97
Wendeu-Foyet	2018	prostate cancer	Never night work	1,088	532
Wendeu-Foyet	2018	prostate cancer	Ever night work	605	286
Behrens	2017	prostate cancer	0–<1 year of shift work	1,156	38
Behrens	2017	prostate cancer	Ever ≥1 years of shift work	601	38
Dickerman	2016	prostate cancer	day shift	9,218	509
Dickerman	2016	prostate cancer	night shift	94	2
Dickerman	2016	prostate cancer	rotating shift	1,771	80
Papantoniou	2014	prostate cancer	Never night work	1717	733
Papantoniou	2014	prostate cancer	Ever night work	766	362
**Sum**				**17,891**	**3,045**
Hulsegge	2018	diabetes	Never shift workers	808	-
Hulsegge	2018	diabetes	Former shift workers	128	-
Hulsegge	2018	diabetes	Current shift workers	125	-
Vetter	2018	diabetes	Day workers	224,928	5,173
Vetter	2018	diabetes	Shift workers, but only rarely, if ever night shifts	23,172	730
Vetter	2018	diabetes	Irregular or rotating shifts with some night shifts	13,559	461
Vetter	2018	diabetes	Irregular or rotating shifts with usual night shifts	3,754	169
Vetter	2018	diabetes	Permanent night shifts	6,801	237
Vetter	2015	diabetes	Non-rotating night shift work	18,638	77
Vetter	2015	diabetes	Rotating night shift work	44,305	242
**Sum**				**336 ,218**	**-**
Liu	2023	mental health	No shift work	183,974	6,224
Liu	2023	mental health	Evening/weekend shifts	18,079	851
Liu	2023	mental health	Irregular night shifts	10,656	476
Liu	2023	mental health	Permanent night shifts	7,942	351
Behrens-men	2021	mental health	Shift work never/ <1 year	220	11
Behrens-men	2021	mental health	Shift work ever	75	8

Behrens-women	2021	mental health	Shift work never/ <1 year	168	24
Behrens-women	2021	mental health	Shift work ever	23	4
Cheng	2021	mental health	Day work	4,973	1,054
Cheng	2021	mental health	Shift work without night shifts	1,419	319
Cheng	2021	mental health	Shift work with night shifts	3,997	815
Cheng	2021	mental health	Fixed night work	248	54
**Sum**				**568,844**	**2,128**

Supplement Table S2 **Exposure and outcome measurements of the included studies**

NO.	Study ID	Covariates adjusted	Shift work exposure/Chronotype measurement	Work schedule	Outcome measurements
**Breast cancer**					
1	Schernhammer-2022	Age, smoking status (never, occasional, former, current), body mass index (BMI, kg/m^2), physical activity (leisure-time metabolic equivalents METs, quintiles; use of oral contraceptives (yes/no), alcohol consumption (number of drinks per day on average, with one standard drink defined as 12 g of alcohol, based on reported weekly or monthly consumption of beer, wines or spirits), educational status (<6 years, 6 years, middle school, high school or more), socioeconomic status i.e. social class (upper white collar, lower white collar, skilled worker, unskilled worker, farmer, other), and zygosity (MZ, DZ, XZ)	**Rotating-shift work:** work that rotated through morning, evening, or night shifts in either a two-shift or three-shift pattern.<br>**Shift work measurement**: information was queried by assessing the respondent's current or latest work type. The question "The present work or the work you last did (mainly) is regular day work, regular night work, two-shift work without a night shift, two-shift work with a night shift, three-shift work, or never worked".<br>**Chronotype measurement**: Chronotype was assessed by a question according to the **Diurnal Type Scal**e "Will you try to estimate to what extent your being 'a morning or an evening people?". It is akin to item 19 on the Horne and Østberg morningness-eveningness questionnaire (MEQ).<br><br>Four categories<br>Definite morning type<br>Somewhat morning type<br>Somewhat evening type<br>Definite evening type	1. Fixed days only<br>2. Rotating 2-shifts without night work<br>3. Rotating 2- or 3-shifts with night work or fixed nights	Data on breast cancer incidence (ICD-10 code 174) were obtained through record linkage (using unique personal identity codes assigned to every permanent resident of Finland) to the Finnish Cancer Registry, where 100% of registered cases are histologically verified.
2	Papantoniou, 2016<br>Spain	Age, educational level, family socioeconomic level, race, BMI, family history of breast cancer, age of menarche, parity, age at the first birth, menopausal status, smoking status, oral contraceptive use and history of hormonal replacement therapy, leisure time physical activity information, current sleep duration and sleep problems, diet habits as well as current and past alcohol consumption	**Night Shift Work definition**: Night work was defined as a working schedule that involved partly or entirely working **between 00:00 and 6:00 a.m.** at least three nights per month. This definition included overnight, late evening (end after 00:00) and early morning (start before 6:00) shifts.<br>**Night Shift Work Assessment**: Detailed questions were used to ascertain information on shift work for each job, including shift work type (permanent vs. rotating), beginning and ending year, time schedules, hours worked per day, job title and workers' activity.<br>**Chronotype measurement**: MCTQ estimated the mid-sleep time on free days corrected for oversleep on free days compared to working days.<br>Three categories<br>Morning type: MSF < 04:00 hr<br>Neither type: MSF = 04:01- 05:00 hr<br>Evening type: MSF > 05:00 hr	1. Ever night shift work (ENW):<br>1.1 Permanent night shift work (ENW1)<br>1.2 Rotating night shift work (ENW2)<br>2. Never night work (NNW): never worked at night including permanent day workers, shift workers with less than three nights per month and women who had never worked	Medical records including tumor hormonal receptor status, differentiation grade and histological type.

3	Hansen, 2012<br>Denmark	Age in 5-year groups at the date of completion of the questionnaire (<50, 50-54, 55-59, 60-64, 65-69 and 70-75 years), length of education, body mass index, alcohol drinking, menopausal status, use of hormone replacement therapy, use of contraceptives, occupational exposure to radar or EMF, occupational physical activity, satisfactory influence on job, too high a workload and work pace, age at menarche, age at menopause, number of child births, tobacco smoking and occasional sun exposure	**Night Shift Work definition**: Night shift work was defined as working for at least 1 year during hours beginning **after 17:00 and ending before 9:00**, not including overtime.<br>**Shift Work Assessment**: structured questionnaire elicited information on all jobs held for at least 1 year and on specific exposures of night shift work.<br>**Chronotype measurement**: diurnal preference (morning, evening, neither).	1. Night shift work (ENW)<br>1.1 Permanent night shift work<br>1.2 Rotating night shift work<br>2. Never night work (NNW)	Information from Danish Cancer Registry
**Prostate cancer**					
4	Lozano-Lorca, 2020<br>Spain	Age, education, first-grade family history of Pca (prostate cancer), physical activity, and smoking status	**Night Shift Work definition**: working partly or entirely (≥3 h) between **22:00 and 06:00**, at least three times per month.<br>The reference group consisted of men who had never performed night shift work for at least a year. Rotating shift workers with no night shifts were included in the reference group (never night shift).<br>**Shift Work Assessment:** Face-to-face interviews: Occupational history data were obtained for all jobs that lasted at least a year including company, location, tasks involved, start and stop dates, and shift work.<br>**Chronotype measurement:** MCTQ at 40 years old.<br>Chronotype were categorized as: (i) morning type: MSFsc <04:00 a.m.; (ii) neither type: MSFsc ≥4:00–≤5:00 a.m.; and (iii) evening type: MSFsc >05:00 a.m.	1. Ever night shift work (SWP1)<br>2. Permanent night work (SWP2)<br>3. Rotating shift work (SWP3)<br>4. Never night shift (NNW)	Histological confirmation: ICD-10. Clinical information of PCa cases were extracted from medical records including Gleason score and stage at diagnosis
5	Wendeu-Foyet, 2018<br>France	Age, ethnic origin and family history of prostate cancer, educational level, body mass index, physical activity and sleep duration	**Night Shift Work definition**: who performed at least 270 hours of night work per year or three nights per month during at least 1 year, according to the French legal definition. Night work was defined as early morning shifts (shift starting between midnight and 06:00), late evening shifts (shift ending between 21:00 and 02:00) and overnight shifts (shift starting before 00:00 and ending after 05:00).<br>**Shift Work Assessment**: participants were asked if they had a non-day schedule for each given job. For each job for which a non-day schedule was indicated, they completed a specific 'night work' questionnaire gathering detailed information on their work time	1. Ever night work (SWP1)<br>1.1 Early morning<br>1.2 Late evening<br>1.3 Overnight shifts<br>2. Permanent night work (SWP2)<br>3. Rotating night work (SWP3)<br>4. Never night work (NNW)	Medical records: prostatic specific antigen (PSA) levels, Gleason Score and stage at diagnosis

			schedule, including lifetime cumulative duration of night work, number of consecutive nights, night shift length, and lifetime cumulative number of nights according to the median values<br>**Chronotype measurement**: Use MEQ to classify cases and controls as a morning, evening or undifferentiated persons		
6	Behrens, 2017<br>Germany	Age, smoking status, body mass index, physical activity, alcohol consumption, family history of prostate cancer, school education, income	**Shift Work definition**: Shift work was defined as any regular employment in shift systems including work hours outside 07:00–18:00 hours, whereas night work was defined as a shift that included work between **24:00–05:00** hours.<br>**Shift Work Assessment**: baseline survey solicited employment in shift work from all subjects who had been gainfully employed or unemployed for less than two years: (1) "Have you ever been employed in shift work?" (never; yes, not involving night work; yes, involving night work), and (2) "How long have you been involved in shift work?". Follow-up interview included a detailed phase-by-phase assessment of the shift-work history and specific exposure characteristics.<br>**Chronotype measurement**: follow-up interview solicited the preferred bed- and wake-up time during days off work to calculate the preferred midpoint of sleep. Early, intermediate, and late preferred sleep midpoint, resulting in age-specific sleep midpoints <02:30-03:00 hours for the early, 02:30-04:00 hours for the intermediate, and >03:30–04:00 hours for the late preference.	1. Ever shift work (ESW)<br>2. Ever night shift work (ENS)	An independent endpoint committee of the HNR study evaluated patient records and, in the case of deceased participants, death certificates to assess the validity of the incident endpoints. Agreement of questionnaire-based incident cancer cases with medical records was evaluated for an 8-year follow-up.
7	Dickerman, 2016<br>Finland	Age, education (<6 years, 6 years, middle school, high school or more), BMI (kg/m2), physical activity (sedentary, occasional exerciser, conditioning exerciser), social class (upper white collar, lower white collar, skilled worker, unskilled worker, farmer, other), smoking status (never, occasional, former, current), alcohol use (deciles of number of drinks per week, with one standard drink defined as 12 grams of alcohol), snoring (never, sometimes, often, nearly always), and zygosity (MZ, DZ, XZ)	**Shift Work definition**: Rotating-shift work refers to work that rotated through morning, evening, or night shifts in either a two-shift or three-shift pattern.<br>**Shift Work Assessment**: assessing the respondent's current or latest work type and were classified into 4 categories: fixed days, fixed nights, rotating shift, and not recently working. Rotating-shift work refers to work that rotated through morning, evening, or night shifts in either a two-shift or three-shift pattern.<br>**Chronotype measurement**: using one question: "Will you try to estimate to what extent you are a morning or an evening person?" The response categories for chronotype included: "I am clearly 'a morning person' (morning spry and evening sleepy)," "I am to some extent 'a morning person,'" "I am to some extent 'an evening person' (morning sleepy and evening spry)," "I am clearly 'an evening person'". We classified chronotype data into four categories: definite	1. Fixed days<br>2. Fixed nights<br>3. Rotating shift:<br>2-shift pattern<br>3-shift patter<br>4. Not recently working	Histologically verified

			morning type, somewhat morning type, somewhat evening type, and definite evening type.		
8	Papantoniou, 2014<br>Spain	Age, educational level, family socioeconomic level, race, body mass index (BMI), family history of prostate cancer, smoking status and leisure time physical activity, food consumption	**Night Shift Work definition**: Night work was defined as a working schedule that involved partly or entirely working **between 00:00 and 6:00 a.m.** at least three nights per month. This definition included overnight late evening (end after 00:00) and early morning (start before 6:00) shifts.<br>**Night Shift Work Assessment**: Detailed questions were used to ascertain information on shift work for each job, including shift work type (permanent vs. rotating), beginning and ending year, time schedules, hours worked per day, job title and workers' activity.<br>**Chronotype measurement**: MCTQ estimated the mid-sleep time on free days corrected for oversleep on free days compared to working days.<br>Three categories<br><u>Morning type</u>: MSF < 04:00 hr<br><u>Neither type</u>: MSF = 04:01– 05:00 hr<br><u>Evening type</u>: MSF > 05:00 hr	SWP<br>1. Ever night work (ENW, including late evening, overnight and early morning shifts):<br>1.1 Permanent night shift work (ENW1)<br>1.2 Rotating night shift work (ENW2)<br>2. Never night work (NNW): Rotating shift without nights	Doctor diagnosis
**Diabetes mellitus**					
9	Hulsegge, 2018<br>Netherlands	Educational attainment, lifestyle and use of anti-hypertensive medication and cholesterol-lowering medication	**Shift Work definition**: evening shifts (i.e., shifts ending before midnight), night shifts (i.e., shifts that continued or started after midnight)<br>**Shift Work Assessment**: Shift work status at each wave was assessed retrospectively in 2013-15 using a questionnaire based on the most important aspects of shift work.<br>**Chronotype measurement**: In 2013-15, chronotype was assessed by a single question: 'How would you describe yourself?'<br><u>Morning type</u>: 'definitely a morning type' OR 'rather more a morning than an evening type'<br><u>Evening type</u>: 'definitely an evening type' OR 'rather more an evening than a morning type'<br><u>Intermediate type</u>: neither	1. Shift work<br>2. Former shift work<br>3. Never shift work<br>4. Day work	1. Cardiometabolic risk factor: Weight, height, total and HDL cholesterol, diastolic and systolic blood pressure, self-reported diabetes (i.e., 11.1 mmol/l) measured at waves 2-6.<br>2. Standardized enzymatic methods were used to retrospectively determine triglycerides, γ-glutamyl-transferase (GGT), high sensitivity CRP, uric acid, cystatin C and creatinine of waves 2-5 using blood plasma.<br>3. Estimated glomerular filtration rate (eGFR) was estimated with the Chronic Disease Epidemiology Collaboration (CKD-EPI) equation

10	Vetter, 2018<br>U.K	Age, sex, ethnicity, family history of diabetes, the Townsend Deprivation Index, BMI, physical activity, smoking, alcohol consumption, sleep apnea, self-reported depressive symptoms, hypertension status, antihypertensive medication use, elevated cholesterol levels, cholesterol-lowering medication use, and statin and steroid use	**Shift Work definition**: A schedule falling outside of **9:00 A.M. to 5:00 P.M.**; by definition, such schedules involved afternoon, evening, or night shifts or rotating through these shifts.<br>**Shift Work Assessment:** Participants employed <u>at baseline</u> were asked to report whether their <u>current main job</u> involved shift work. If yes, participants were further asked whether their main job involved night shifts. In the lifetime employment assessment, individual number of years working night shifts, the average number of night shifts per month and cumulative lifetime night shift exposure were recorded.<br>**Chronotype measurement:** Self-reported chronotype <u>at baseline</u> by answering a question taken from the **<u>MEQ</u>**: i. Definitely a 'morning' person; ii. More a 'morning' than 'evening' person; iii. More an 'evening' than a 'morning' person; iv. Definitely an 'evening' person; v. Do not know; vi. Prefer not to answer	1. Shift workers, but only rarely, if ever night shifts (SWP1)<br>2. Irregular or rotating shifts with some night shifts (SWP2)<br>3. Irregular or rotating shifts with usual night shifts (SWP3)<br>4. Permanent night shifts (SWP4)<br>5. Day work	1. Polygenic Risk Score (GRS): A second GRS (GRS10) was derived from a subset of 10 SNPs with a reported **OR >1.2 for type 2 diabetes**.<br>2. Ascertainment of Cases of Type 2 Diabetes: self- reported and trained health professional queried medical history and medication use at baseline to derive case status
11	Vetter, 2015<br>U.S.A	Age, body weight, cigarette smoking, family history of diabetes, physical activity, menopausal status, and hormone intake, annual household income, alcohol consumption, average sleep duration, potential depressive symptoms	**Shift Work definition**: At least 3 nights/month in addition to other days and evenings in that month<br>**Shift Work Assessment:** women indicated in 1989 how many years of <u>rotating night shift work</u> they had worked until then, with updates in 1991, 1993, 1997, 2001, 2005, and 2009, and retrospective rotating night shift work assessments for 1997–1999 (in 2001), 2001–2003 (in 2005), and 2005–2007 (in 2009) were included in the next biennial questionnaire.<br>**Chronotype measurement:** a question from the **<u>MEQ,</u>** "One hears about 'morning' and 'evening' types of people. Which one of these types do you consider yourself to be?"<br><u>MT</u>: definitely a morning type<br><u>IT</u>: rather more a morning than an evening type + rather more an evening than a morning type + neither<br><u>ET</u>: definitely an evening type	1. Rotating night shift work (RNS)<br>2. No rotating night shift work (NRNS)	1. Self-reported diagnosis of diabetes with supplemental information regarding symptoms, diagnostic tests, and hypoglycemic therapy.<br>2. A case of T2D was considered confirmed if one of the following National Diabetes Data Group criteria
**Mental health**					
12	Liu-2023	age, sex, ethnic, socioeconomic status (SES), body mass index (BMI), household income before tax per year, study center region, education group, smoking status, alcohol drinker status, mental consulting from a psychiatrist or general practitioner (GP) for nerves,	**Shift Work definition**: A schedule falling outside of **9:00 A.M. to 5:00 P.M.**; by definition, such schedules involved afternoon, evening, or night shifts or rotating through these shifts.<br>**Shift Work Assessment:** Participants employed <u>at baseline</u> were asked to report whether their <u>current main job</u> involved shift work. If yes, participants were further asked whether their main job involved night shifts. In the lifetime employment assessment, individual	1. Shift workers, but only rarely, if ever night shifts (SWP1)<br>2. Irregular or rotating shifts with some night shifts (SWP2)<br>3. Irregular or rotating shifts with usual night shifts (SWP3)	Incident depression from clinical diagnoses was identified from the part of mental and behavioral disorders in the "first occurrence fields" of UKB (data category: 2405), which included data from primary care, hospital inpatient

		anxiety, tension or depression, total physical activity, and self-reported overall health	number of years working night shifts, the average number of night shifts per month and cumulative lifetime night shift exposure were recorded.<br>**Chronotype measurement:** Self-reported chronotype at baseline by answering a question taken from the **MEQ**: i. Definitely a 'morning' person; ii. More a 'morning' than 'evening' person; iii. More an 'evening' than a 'morning' person; iv. Definitely an 'evening' person; v. Do not know; vi. Prefer not to answer	4. Permanent night shifts (SWP4)<br>5. Day work	record, self-reported medical condition, and death registers. The codes of F32 (depressive episode) and F33 (recurrent depressive disorder) from the International Classification of Diseases, 10th Revision (ICD-10) were used as incident depression.
13	Behrens, 2021 Germany	Age, years of school education, cohabitation (with partner or children versus single) and social network, body mass index (kg/m2), a categorical co-morbidity index indicating the presence of myocardial infarction, cardiac insufficiency, stroke, diabetes, chronic bronchitis, asthma, atopic dermatitis, psoriasis, cancer, rheumatism, herniated vertebral disc or migraine, smoking habits, and a family history of psychiatric disease, poor sleep quality, a dramatic life event during the last 6 months, the person's prevailing mood	**Shift Work definition**: any regular employment in shift systems including work hours outside 7:00 to 18:00, whereas night work was defined as shift work shifts entailing work between midnight and 5:00.<br>**Shift Work Assessment**: At baseline, shift work information was collected for subjects who were currently employed or unemployed for less than 2 years using two simple questions: 1) "Have you ever worked in shift work?" with the response categories "no; yes; without night shifts; yes, with night shifts," and 2) "How long have you worked in shift work?" in months and years. In the 10-year follow-up survey, detailed phase-by-phase questions on the subjects' shift- and night-work history was asked.<br>**Chronotype measurement**: assessed each subjects' diurnal preference as proxy for the individual chronotype as early, intermediate and late midpoint of sleep at the 10-year follow-up.	1. Ever shift work (SWP1)<br>2. Ever night work (SWP2)<br>3. Day work	1. The Center for Epidemiologic Studies Depression Scale (CES-D): A sum score of ≥17.<br>2. Patient Health Questionnaire (PHQ)<br>3. Self-reported prescription of an anti-depressant
14	Cheng, 2021 Finland	Age, gender, sleep problems, job demands and working hours.	**Shift Work definition**: Not defined. Day work, shift work without night shifts, shift work with night shifts, fixed night work, or other irregular work.<br>**Shift Work Assessment**: the participants self- reported their current work schedule either as day work, shift work without night shifts, shift work with night shifts, fixed night work, or other irregular work.<br>**Chronotype measurement**: Chronotype was assessed using one item from the Diurnal Type Scale in the 2015 survey: "Do you think you are a morning person or an evening person?" The respondents chose between definite morning type, somewhat morning type, somewhat evening type, or definite evening type.	1. Shift work without night shifts (SWP1)<br>2. Shift work with night shifts (SWP2)<br>3. Fixed night work (SWP3)<br>4. Day work	1. The presence of mood disorders was detected using the GHQ-12. The answers were dichotomized as no distress for 0 and 1 and distressed for 2 and 3. Those who had ≥4 distressed responses were categorized as experiencing mood disorders. A cutoff GHQ-12 score of 3 or 4 has been recommended to screen psychiatric patients from the general population.

Abbreviation

MCTQ: Munich ChronoType Questionnaire; MEQ: Morningness-Eveningness Questionnaire; DTS: Diurnal Type Scale; CES-D: The Center for Epidemiologic Studies Depression Scale; PHQ-9: Patient Health Questionnaire; GHQ-12: General Health Questionnaire.

Supplementary Table S3 Exposure-response relationships between cumulative years/nights and breast /prostate cancer.

	β	SE	p value	β	SE	p value
Breast cancer	Cumulative years of night shifts [a]			Cumulative nights of shifts [b]		
Morning type	-0.002	0.009	0.832	0.000	0.001	0.390
Intermediate type	0.012	0.011	0.243	0.000	0.000	0.204
Evening type	0.020	0.013	0.123	0.000	0.000	0.092
Prostate cancer	Cumulative years of night shifts [c]			Cumulative nights of shifts [d]		
Morning type	0.006	0.005	0.265	0.000	5.09E-05	0.479
Intermediate type	-0.001	0.005	0.883	3.29E-05	4.87E-05	0.499
Evening type	**0.021**	**0.008**	**0.012**	0.000	0.000	0.115

Notes

[a] Studies included (breast cancer):

Papantoniou-2016, cumulative years of night shift category: Never night work; 1-4 years; 5-14 years; >=15 years.

[b] Studies included (breast cancer):

Papantoniou-2016, cumulative nights of shifts: Never night work; 36–599 nights; 600–1799 nights; >=1800 nights.

Hansen-2012, cumulative nights of shifts: Never night work; <884 nights; >=884 nights.

[c] Studies included (prostate cancer):

Lozano-Lorca-2020, cumulative years of night shift category: Never night work; ≤7 years; 7–≤26 years; >26 years.

Wendeu-Foyet-2018, cumulative years of night shift category: Never night work; <10 years; 10-19 years; 20-29 years; ≥30 years.

Behrens-2017, cumulative years of night shift category: 0-<1 year; 1-<10 years; ≥10 years.

[d] Studies included (prostate cancer):

Wendeu-Foyet-2018, cumulative nights of shifts: Never night work; <1314 nights; ≥1314 nights.

Papantoniou-2014, cumulative nights of shifts: Never night work; <= 1152 nights; 1153–2856 nights; >=2857 nights.

Supplementary Figure S1 Risk of bias assessment

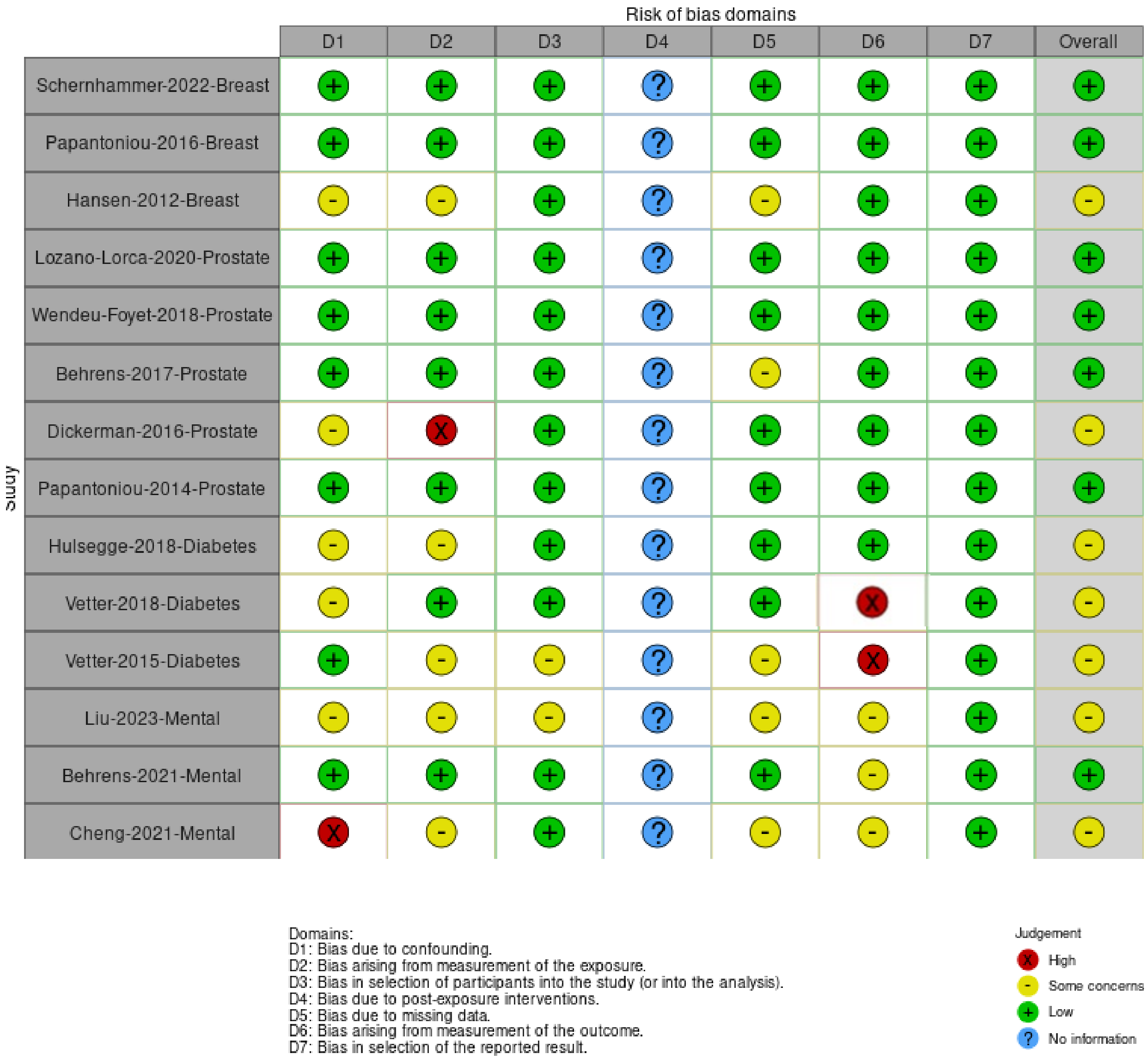

Supplementary Figure S2 Shift work by chronotype and diseases

(a) Breast cancer

Author- Study year	Weight	OR	95% CI
**chronotype = Morning**			
Schernhammer 2022 2-shifts without nights	9.7%	0.91	[0.56; 1.49]
Schernhammer 2022 3-shifts or nights only	11.7%	1.46	[0.93; 2.29]
Papantoniou 2016 Ever night work	19.9%	1.17	[0.83; 1.65]
Hansen 2012 <884 nights	2.3%	1.30	[0.48; 3.54]
Hansen 2012 >=884 nights	3.0%	3.90	[1.60; 9.50]
**Total (95% CI)**	**46.6%**	**1.36**	**[0.94; 1.96]**
Heterogeneity: $Tau^2 = 0.0860$; $Chi^2 = 8.45$, df = 4 (P = 0.08); $I^2 = 53\%$			
**chronotype = Evening**			
Schernhammer 2022 2-shifts without nights	7.0%	0.73	[0.41; 1.30]
Schernhammer 2022 3-shifts or nights only	11.3%	1.56	[0.99; 2.46]
Papantoniou 2016 Ever night work	11.5%	1.27	[0.81; 2.00]
Hansen 2012 <884 nights	1.3%	0.80	[0.21; 3.10]
Hansen 2012 >=884 nights	2.1%	2.00	[0.69; 5.76]
**Total (95% CI)**	**33.2%**	**1.21**	**[0.86; 1.70]**
Heterogeneity: $Tau^2 = 0.0444$; $Chi^2 = 5.37$, df = 4 (P = 0.25); $I^2 = 25\%$			
**chronotype = Intermediate**			
Papantoniou 2016 Ever night work	18.0%	1.17	[0.81; 1.68]
Hansen 2012 <884 nights	1.4%	1.00	[0.27; 3.65]
Hansen 2012 >=884 nights	0.8%	0.70	[0.13; 3.83]
**Total (95% CI)**	**20.2%**	**1.13**	**[0.81; 1.59]**
Heterogeneity: $Tau^2 = 0$; $Chi^2 = 0.37$, df = 2 (P = 0.83); $I^2 = 0\%$			
**Total (95% CI)**	**100.0%**	**1.23**	**[1.05; 1.43]**

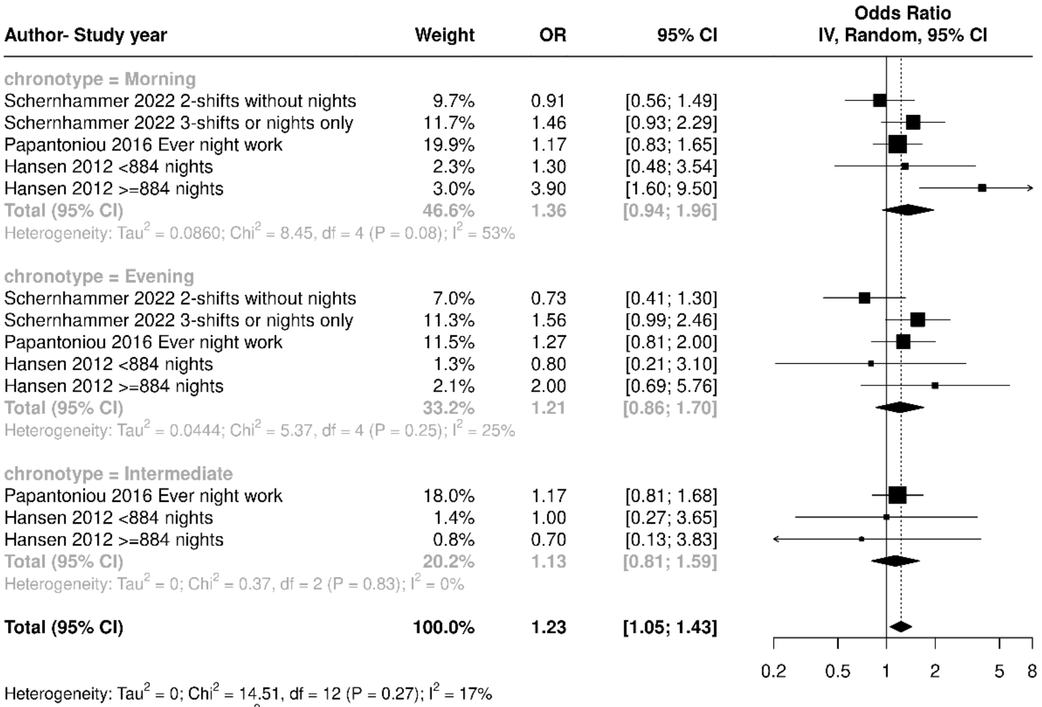

Heterogeneity: $Tau^2 = 0$; $Chi^2 = 14.51$, df = 12 (P = 0.27); $I^2 = 17\%$
Test for subgroup differences: $Chi^2 = 0.52$, df = 2 (P = 0.77)

(b) Prostate cancer

Author- Study year	Weight	OR	95% CI
**chronotype = Morning**			
Lozano-Lorca 2020 Ever night work	7.5%	1.25	[0.78; 2.00]
Wendeu-Foyet 2018 Ever night work	9.1%	0.77	[0.54; 1.10]
Behrens 2017 Ever, >=1 year of shift work	2.0%	5.47	[1.45; 20.67]
Dickerman 2016 rotating shift	8.8%	1.00	[0.68; 1.46]
Papantoniou 2014 Ever night work	10.3%	1.14	[0.87; 1.50]
**Total (95% CI)**	**37.6%**	**1.06**	**[0.85; 1.33]**
Heterogeneity: $Tau^2 = 0.0205$; $Chi^2 = 9.77$, df = 4 ($P = 0.04$); $I^2 = 59\%$			
**chronotype = Intermediate**			
Lozano-Lorca 2020 Ever night work	4.9%	1.71	[0.83; 3.52]
Wendeu-Foyet 2018 Ever night work	5.8%	0.96	[0.51; 1.79]
Behrens 2017 Ever, >=1 year of shift work	5.7%	2.37	[1.26; 4.45]
Dickerman 2016 rotating shift	6.0%	0.50	[0.27; 0.91]
Dickerman 2016 rotating shift	8.6%	1.50	[1.01; 2.22]
Papantoniou 2014 Ever night work	9.2%	1.02	[0.72; 1.44]
**Total (95% CI)**	**40.1%**	**1.19**	**[0.79; 1.79]**
Heterogeneity: $Tau^2 = 0.1832$; $Chi^2 = 16.05$, df = 5 ($P < 0.01$); $I^2 = 69\%$			
**chronotype = Evening**			
Lozano-Lorca 2020 Ever night work	2.2%	3.14	[0.91; 10.80]
Wendeu-Foyet 2018 Ever night work	6.5%	1.83	[1.05; 3.19]
Behrens 2017 Ever, >=1 year of shift work	1.6%	1.20	[0.27; 5.31]
Dickerman 2016 rotating shift	5.6%	1.50	[0.79; 2.86]
Papantoniou 2014 Ever night work	6.3%	1.50	[0.85; 2.65]
**Total (95% CI)**	**22.2%**	**1.67**	**[1.21; 2.29]**
Heterogeneity: $Tau^2 = 0$; $Chi^2 = 1.54$, df = 4 ($P = 0.82$); $I^2 = 0\%$			
**Total (95% CI)**	**100.0%**	**1.25**	**[1.02; 1.53]**

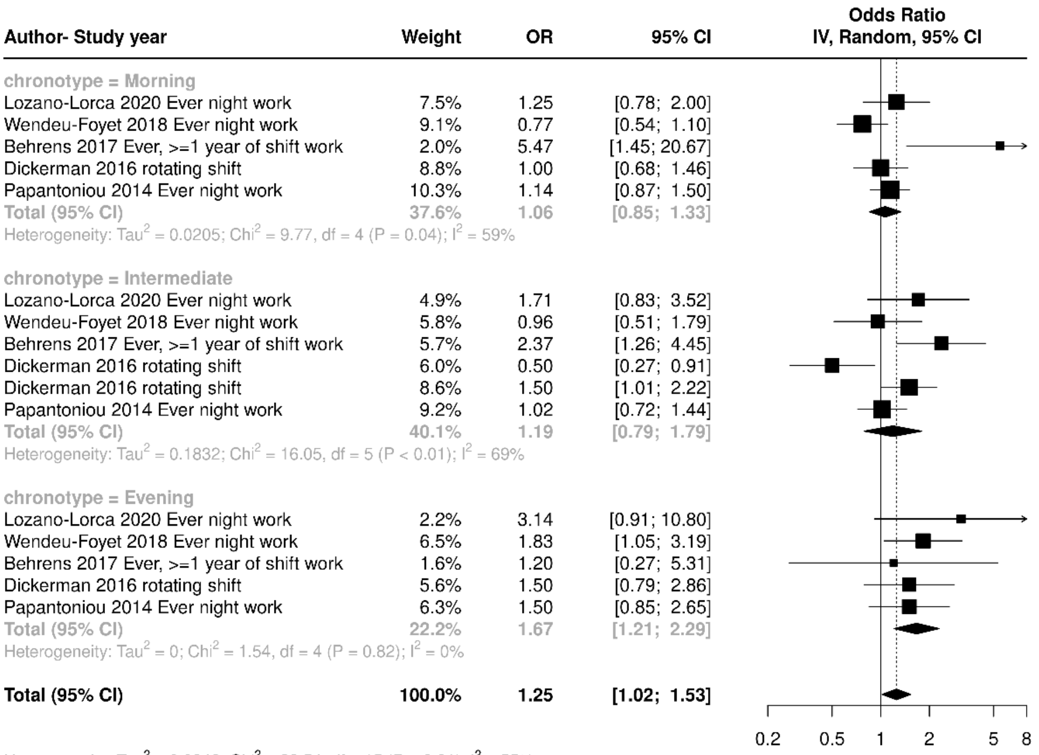

Heterogeneity: $Tau^2 = 0.0848$; $Chi^2 = 33.54$, df = 15 ($P < 0.01$); $I^2 = 55\%$
Test for subgroup differences: $Chi^2 = 5.06$, df = 2 ($P = 0.08$)

(c) Diabetes

Author- Study year	Weight	OR	95% CI
chronotype = Morning			
Hulsegge 2018 current shift workers	0.1%	5.50	[0.86; 35.14]
Vetter 2018 Shift workers, but only rarely, if ever night shifts	12.5%	1.15	[0.96; 1.37]
Vetter 2018 Irregular or rotating shifts with some night shifts	7.9%	1.07	[0.86; 1.34]
Vetter 2018 Irregular or rotating shifts with usual night shifts	2.3%	1.14	[0.75; 1.73]
Vetter 2018 Permanent night shifts	3.0%	0.86	[0.60; 1.24]
Vetter 2015 Non-rotating night shift work	1.4%	0.75	[0.44; 1.28]
Vetter 2015 RNS: <10 yrs	4.1%	0.91	[0.67; 1.24]
Vetter 2015 >= 10 yrs	0.8%	1.63	[0.79; 3.35]
Total (95% CI)	32.0%	1.06	[0.95; 1.19]
Heterogeneity: $Tau^2 = < 0.0001$; $Chi^2 = 9.11$, df = 7 (P = 0.25); $I^2 = 23\%$			
chronotype = Intermediate			
Hulsegge 2018 current shift workers	0.1%	0.97	[0.11; 8.36]
Vetter 2018 Shift workers, but only rarely, if ever night shifts	24.4%	1.14	[1.00; 1.29]
Vetter 2018 Irregular or rotating shifts with some night shifts	15.0%	1.07	[0.91; 1.26]
Vetter 2018 Irregular or rotating shifts with usual night shifts	5.6%	1.40	[1.07; 1.83]
Vetter 2018 Permanent night shifts	7.4%	1.21	[0.96; 1.53]
Total (95% CI)	52.5%	1.15	[1.06; 1.26]
Heterogeneity: $Tau^2 = 0$; $Chi^2 = 3.08$, df = 4 (P = 0.54); $I^2 = 0\%$			
chronotype = Evening			
Hulsegge 2018 current shift workers	0.2%	1.20	[0.29; 4.99]
Vetter 2018 Shift workers, but only rarely, if ever night shifts	4.1%	1.11	[0.81; 1.52]
Vetter 2018 Irregular or rotating shifts with some night shifts	3.2%	1.55	[1.09; 2.21]
Vetter 2018 Irregular or rotating shifts with usual night shifts	1.4%	1.53	[0.89; 2.62]
Vetter 2018 Permanent night shifts	2.8%	0.99	[0.68; 1.44]
Vetter 2015 Non-rotating night shift work	1.1%	1.43	[0.78; 2.64]
Vetter 2015 RNS: <10 yrs	2.2%	0.86	[0.57; 1.31]
Vetter 2015 >= 10 yrs	0.5%	1.01	[0.43; 2.37]
Total (95% CI)	15.5%	1.17	[0.98; 1.41]
Heterogeneity: $Tau^2 = 0.0120$; $Chi^2 = 6.85$, df = 7 (P = 0.44); $I^2 = 0\%$			
**Total (95% CI)**	**100.0%**	**1.13**	**[1.06; 1.20]**

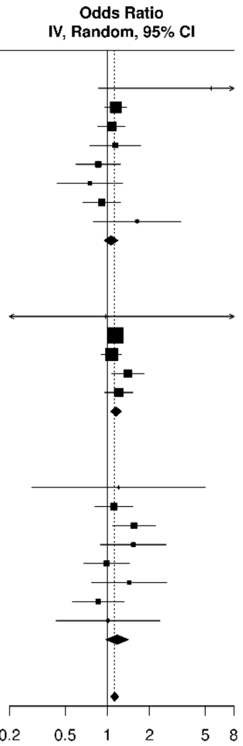

Heterogeneity: $Tau^2 = 0$; $Chi^2 = 20.65$, df = 20 (P = 0.42); $I^2 = 3\%$
Test for subgroup differences: $Chi^2 = 1.57$, df = 2 (P = 0.46)

(d) Poor mental health

Author- Study year	Weight	OR	95% CI
**chronotype = Morning**			
Liu 2023 Evening/weekend shifts	14.8%	1.20	[1.10; 1.31]
Liu 2023 Irregular night shifts	9.9%	1.18	[1.04; 1.34]
Liu 2023 Permanent night shifts	7.7%	1.25	[1.08; 1.45]
Behrens-men 2021 Ever >=1 year shift wor	0.1%	0.50	[0.08; 3.05]
Behrens-women 2021 Ever >=1 year shift wor	0.2%	1.83	[0.67; 4.99]
Cheng 2021 Shift work without night shifts	1.6%	0.98	[0.68; 1.41]
Cheng 2021 Shift work with night shifts	1.5%	0.95	[0.65; 1.38]
Cheng 2021 Fixed night work	0.2%	1.56	[0.60; 4.06]
**Total (95% CI)**	**36.1%**	**1.19**	**[1.12; 1.27]**
Heterogeneity: $Tau^2 = 0$; $Chi^2 = 4.82$, df = 7 (P = 0.68); $I^2 = 0\%$			
**chronotype = Evening**			
Liu 2023 No shift work	23.4%	1.07	[1.02; 1.13]
Liu 2023 Evening/weekend shifts	11.0%	1.11	[0.99; 1.25]
Liu 2023 Irregular night shifts	8.3%	1.19	[1.03; 1.37]
Liu 2023 Permanent night shifts	7.5%	1.04	[0.89; 1.21]
Behrens-men 2021 Ever >=1 year shift wor	0.0%	17.40	[1.49; 203.24]
Cheng 2021 Shift work without night shifts	1.2%	1.02	[0.67; 1.56]
Cheng 2021 Shift work with night shifts	1.4%	1.75	[1.18; 2.60]
Cheng 2021 Fixed night work	0.5%	2.05	[1.06; 3.97]
**Total (95% CI)**	**53.4%**	**1.11**	**[1.05; 1.17]**
Heterogeneity: $Tau^2 = 0.0009$; $Chi^2 = 16.4$, df = 7 (P = 0.02); $I^2 = 57\%$			
**chronotype = Intermediate**			
Behrens-men 2021 Ever >=1 year shift wor	0.2%	0.67	[0.20; 2.26]
Behrens-women 2021 Ever >=1 year shift wor	0.1%	0.72	[0.10; 5.03]
Cheng 2021 Shift work without night shifts	2.2%	0.98	[0.72; 1.34]
Cheng 2021 Shift work with night shifts	2.2%	0.98	[0.72; 1.34]
Cheng 2021 Fixed night work	0.4%	0.87	[0.39; 1.94]
Cheng 2021 Shift work without night shifts	2.3%	1.35	[1.00; 1.83]
Cheng 2021 Shift work with night shifts	2.6%	1.11	[0.83; 1.48]
Cheng 2021 Fixed night work	0.7%	1.91	[1.09; 3.34]
**Total (95% CI)**	**10.5%**	**1.12**	**[0.97; 1.30]**
Heterogeneity: $Tau^2 = 0.0031$; $Chi^2 = 7.62$, df = 7 (P = 0.37); $I^2 = 8\%$			
**Total (95% CI)**	**100.0%**	**1.14**	**[1.09; 1.20]**

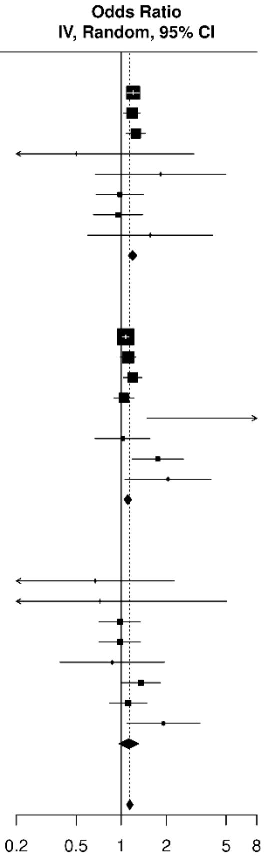

Heterogeneity: $Tau^2 = 0.0019$; $Chi^2 = 33.65$, df = 23 (P = 0.07); $I^2 = 32\%$
Test for subgroup differences: $Chi^2 = 2.97$, df = 2 (P = 0.23)

Supplementary Figure S3 Night shift work by chronotype

(a) Breast cancer

Author- Study year	Weight	OR	95% CI
**chronotype = Morning**			
Schernhammer 2022 3-shifts or nights only	14.0%	1.46	[0.93; 2.29]
Papantoniou 2016 Ever night work	23.9%	1.17	[0.83; 1.65]
Hansen 2012 <884 nights	2.8%	1.30	[0.48; 3.54]
Hansen 2012 >=884 nights	3.6%	3.90	[1.60; 9.50]
**Total (95% CI)**	**44.3%**	**1.54**	**[1.01; 2.37]**
Heterogeneity: $Tau^2 = 0.0944$; $Chi^2 = 6.18$, df = 3 (P = 0.10); $I^2 = 51\%$			
**chronotype = Evening**			
Schernhammer 2022 3-shifts or nights only	13.6%	1.56	[0.99; 2.46]
Papantoniou 2016 Ever night work	13.8%	1.27	[0.81; 2.00]
Hansen 2012 <884 nights	1.5%	0.80	[0.21; 3.10]
Hansen 2012 >=884 nights	2.5%	2.00	[0.69; 5.76]
**Total (95% CI)**	**31.5%**	**1.41**	**[1.04; 1.90]**
Heterogeneity: $Tau^2 = 0$; $Chi^2 = 1.49$, df = 3 (P = 0.69); $I^2 = 0\%$			
**chronotype = Intermediate**			
Papantoniou 2016 Ever night work	21.6%	1.17	[0.81; 1.68]
Hansen 2012 <884 nights	1.7%	1.00	[0.27; 3.65]
Hansen 2012 >=884 nights	1.0%	0.70	[0.13; 3.83]
**Total (95% CI)**	**24.2%**	**1.13**	**[0.81; 1.59]**
Heterogeneity: $Tau^2 = 0$; $Chi^2 = 0.37$, df = 2 (P = 0.83); $I^2 = 0\%$			
**Total (95% CI)**	**100.0%**	**1.33**	**[1.12; 1.57]**

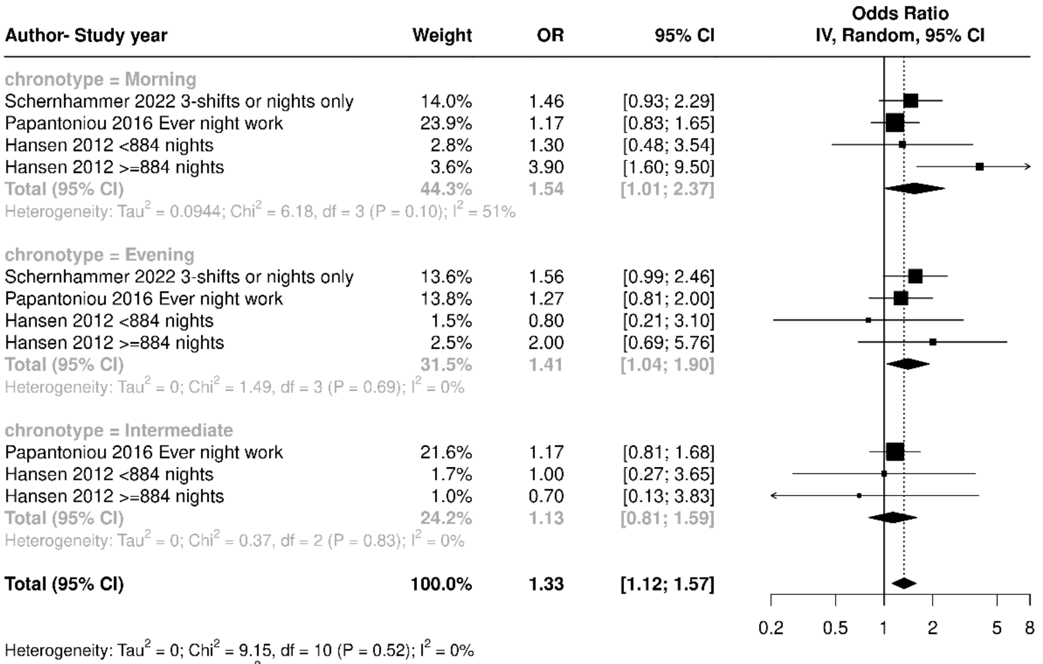

Heterogeneity: $Tau^2 = 0$; $Chi^2 = 9.15$, df = 10 (P = 0.52); $I^2 = 0\%$
Test for subgroup differences: $Chi^2 = 1.44$, df = 2 (P = 0.49)

(b) Prostate cancer

Author- Study year	Weight	OR	95% CI
chronotype = Morning			
Lozano-Lorca 2020 Ever night work	7.9%	1.25	[0.78; 2.00]
Lozano-Lorca 2020 permanent night work	5.4%	0.99	[0.50; 1.95]
Lozano-Lorca 2020 rotating night work	6.2%	1.50	[0.82; 2.75]
Wendeu-Foyet 2018 Ever night work	9.8%	0.77	[0.54; 1.10]
Behrens 2017 1-10 years of night work	1.1%	4.48	[0.74; 27.12]
Behrens 2017 >=10 years of night work	1.8%	7.72	[1.92; 31.00]
Papantoniou 2014 Ever night work	11.1%	1.14	[0.87; 1.50]
Total (95% CI)	43.4%	1.22	[0.90; 1.65]
Heterogeneity: Tau^2 = 0.0751; Chi^2 = 15.18, df = 6 (P = 0.02); I^2 = 60%			
chronotype = Intermediate			
Lozano-Lorca 2020 Ever night work	5.0%	1.71	[0.83; 3.52]
Lozano-Lorca 2020 permanent night work	3.2%	1.64	[0.61; 4.40]
Lozano-Lorca 2020 rotating night work	3.2%	1.77	[0.66; 4.74]
Wendeu-Foyet 2018 Ever night work	6.0%	0.96	[0.51; 1.79]
Behrens 2017 1-10 years of night work	4.1%	2.26	[0.98; 5.20]
Behrens 2017 >=10 years of night work	5.0%	2.52	[1.23; 5.18]
Papantoniou 2014 Ever night work	9.9%	1.02	[0.72; 1.44]
Total (95% CI)	36.4%	1.47	[1.06; 2.04]
Heterogeneity: Tau^2 = 0.0713; Chi^2 = 8.84, df = 6 (P = 0.18); I^2 = 32%			
chronotype = Evening			
Lozano-Lorca 2020 Ever night work	2.2%	3.14	[0.91; 10.80]
Lozano-Lorca 2020 permanent night work	1.5%	3.53	[0.76; 16.37]
Lozano-Lorca 2020 rotating night work	1.4%	2.72	[0.54; 13.66]
Wendeu-Foyet 2018 Ever night work	6.8%	1.83	[1.05; 3.19]
Behrens 2017 1-10 years of night work	1.7%	1.42	[0.33; 6.15]
Papantoniou 2014 Ever night work	6.6%	1.50	[0.85; 2.65]
Total (95% CI)	20.2%	1.84	[1.30; 2.61]
Heterogeneity: Tau^2 = 0; Chi^2 = 2.25, df = 5 (P = 0.81); I^2 = 0%			
**Total (95% CI)**	**100.0%**	**1.43**	**[1.17; 1.75]**

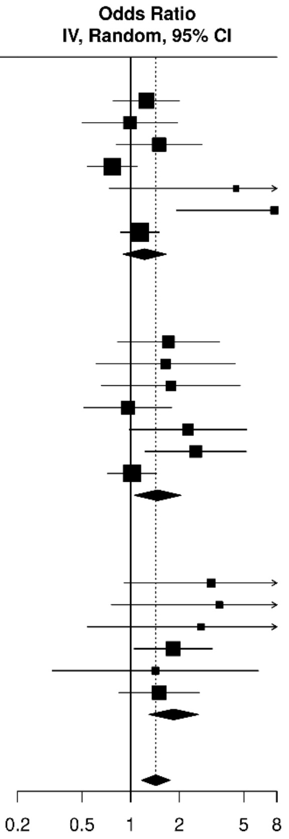

Heterogeneity: Tau^2 = 0.0747; Chi^2 = 32.96, df = 19 (P = 0.02); I^2 = 42%
Test for subgroup differences: Chi^2 = 3.06, df = 2 (P = 0.22)

(c) Diabetes

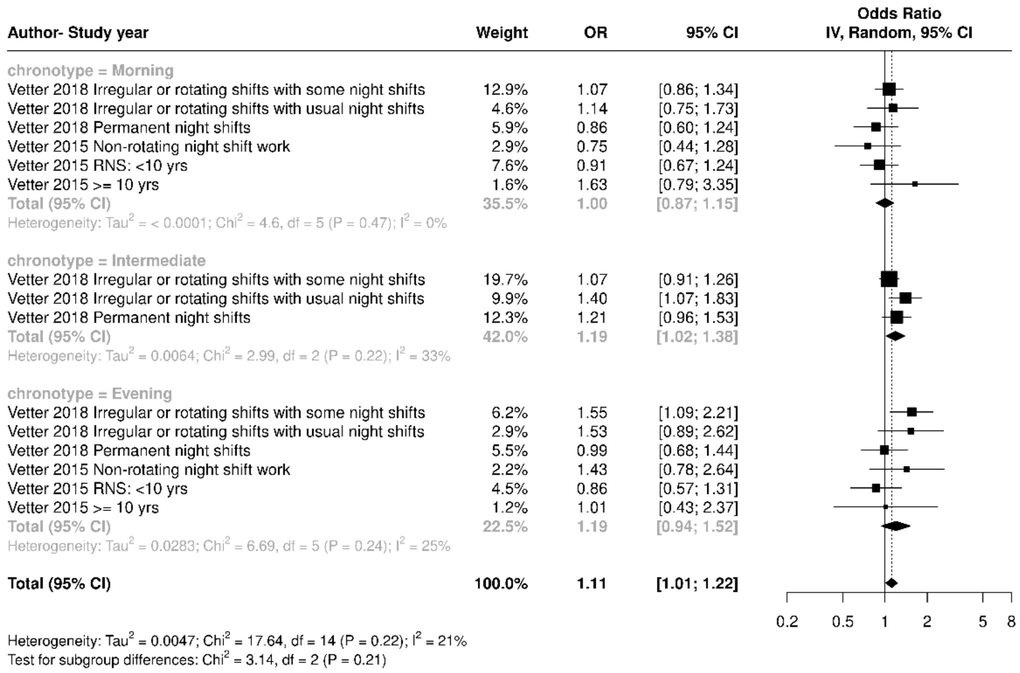

Author- Study year
Weight
OR
95% CI
Odds Ratio
IV, Random, 95% CI
chronotype = Morning
Vetter 2018 Irregular or rotating shifts with some night shifts 12.9% 1.07 [0.86; 1.34]
Vetter 2018 Irregular or rotating shifts with usual night shifts 4.6% 1.14 [0.75; 1.73]
Vetter 2018 Permanent night shifts 5.9% 0.86 [0.60; 1.24]
Vetter 2015 Non-rotating night shift work 2.9% 0.75 [0.44; 1.28]
Vetter 2015 RNS: <10 yrs 7.6% 0.91 [0.67; 1.24]
Vetter 2015 >= 10 yrs 1.6% 1.63 [0.79; 3.35]
Total (95% CI) 35.5% 1.00 [0.87; 1.15]
Heterogeneity: Tau^2 = < 0.0001; Chi^2 = 4.6, df = 5 (P = 0.47); I^2 = 0%
chronotype = Intermediate
Vetter 2018 Irregular or rotating shifts with some night shifts 19.7% 1.07 [0.91; 1.26]
Vetter 2018 Irregular or rotating shifts with usual night shifts 9.9% 1.40 [1.07; 1.83]
Vetter 2018 Permanent night shifts 12.3% 1.21 [0.96; 1.53]
Total (95% CI) 42.0% 1.19 [1.02; 1.38]
Heterogeneity: Tau^2 = 0.0064; Chi^2 = 2.99, df = 2 (P = 0.22); I^2 = 33%
chronotype = Evening
Vetter 2018 Irregular or rotating shifts with some night shifts 6.2% 1.55 [1.09; 2.21]
Vetter 2018 Irregular or rotating shifts with usual night shifts 2.9% 1.53 [0.89; 2.62]
Vetter 2018 Permanent night shifts 5.5% 0.99 [0.68; 1.44]
Vetter 2015 Non-rotating night shift work 2.2% 1.43 [0.78; 2.64]
Vetter 2015 RNS: <10 yrs 4.5% 0.86 [0.57; 1.31]
Vetter 2015 >= 10 yrs 1.2% 1.01 [0.43; 2.37]
Total (95% CI) 22.5% 1.19 [0.94; 1.52]
Heterogeneity: Tau^2 = 0.0283; Chi^2 = 6.69, df = 5 (P = 0.24); I^2 = 25%
Total (95% CI) 100.0% 1.11 [1.01; 1.22]
0.2 0.5 1 2 5 8
Heterogeneity: Tau^2 = 0.0047; Chi^2 = 17.64, df = 14 (P = 0.22); I^2 = 21%
Test for subgroup differences: Chi^2 = 3.14, df = 2 (P = 0.21)

(d) Poor mental health

Author- Study year	Weight	OR	95% CI
chronotype = Morning			
Liu 2023 Evening/weekend shifts	15.8%	1.20	[1.10; 1.31]
Liu 2023 Irregular night shifts	10.8%	1.18	[1.04; 1.34]
Liu 2023 Permanent night shifts	8.5%	1.25	[1.08; 1.45]
Behrens-men 2021 Ever >=1 year night shift wor	0.1%	0.87	[0.11; 7.06]
Behrens-women 2021 Ever >=1 year night shift wor	0.3%	2.33	[0.84; 6.46]
Cheng 2021 Shift work with night shifts	1.8%	0.95	[0.65; 1.38]
Cheng 2021 Fixed night work	0.3%	1.56	[0.60; 4.06]
Total (95% CI)	37.4%	1.20	[1.12; 1.28]
Heterogeneity: $Tau^2 = 0$; $Chi^2 = 3.83$, df = 6 (P = 0.70); $I^2 = 0\%$			
chronotype = Evening			
Liu 2023 No shift work	24.1%	1.07	[1.02; 1.13]
Liu 2023 Evening/weekend shifts	12.0%	1.11	[0.99; 1.25]
Liu 2023 Irregular night shifts	9.2%	1.19	[1.03; 1.37]
Liu 2023 Permanent night shifts	8.2%	1.04	[0.89; 1.21]
Cheng 2021 Shift work with night shifts	1.6%	1.75	[1.18; 2.60]
Cheng 2021 Fixed night work	0.6%	2.05	[1.06; 3.97]
Total (95% CI)	55.7%	1.11	[1.04; 1.17]
Heterogeneity: $Tau^2 = 0.0010$; $Chi^2 = 11.42$, df = 5 (P = 0.04); $I^2 = 56\%$			
chronotype = Intermediate			
Behrens-men 2021 Ever >=1 year night shift wor	0.2%	1.29	[0.40; 4.17]
Behrens-women 2021 Ever >=1 year night shift wor	0.1%	1.21	[0.18; 8.21]
Cheng 2021 Shift work with night shifts	2.5%	0.98	[0.72; 1.34]
Cheng 2021 Fixed night work	0.4%	0.87	[0.39; 1.94]
Cheng 2021 Shift work with night shifts	2.9%	1.11	[0.83; 1.48]
Cheng 2021 Fixed night work	0.8%	1.91	[1.09; 3.34]
Total (95% CI)	6.9%	1.12	[0.93; 1.35]
Heterogeneity: $Tau^2 = 0.0006$; $Chi^2 = 4.63$, df = 5 (P = 0.46); $I^2 = 0\%$			
**Total (95% CI)**	**100.0%**	**1.15**	**[1.09; 1.21]**

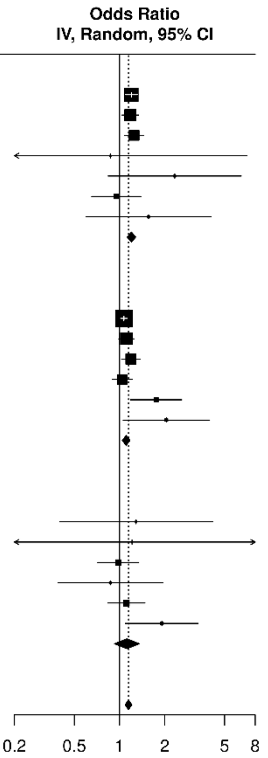

Heterogeneity: $Tau^2 = 0.0022$; $Chi^2 = 25.51$, df = 18 (P = 0.11); $I^2 = 29\%$
Test for subgroup differences: $Chi^2 = 3.35$, df = 2 (P = 0.19)

Supplementary Figure S4 Rotating night shift work by chronotype

(a) Breast cancer

Author- Study year	Weight	OR	95% CI
chronotype = Morning			
Schernhammer 2022 2-shifts without nights	14.0%	0.91	[0.56; 1.49]
Schernhammer 2022 3-shifts or nights only	16.8%	1.46	[0.93; 2.29]
Papantoniou 2016 rotating night work	16.8%	1.11	[0.71; 1.74]
Total (95% CI)	47.7%	1.15	[0.88; 1.51]
Heterogeneity: Tau^2 = < 0.0001; Chi^2 = 1.98, df = 2 (P = 0.37); I^2 = 0%			
chronotype = Evening			
Schernhammer 2022 2-shifts without nights	10.0%	0.73	[0.41; 1.30]
Schernhammer 2022 3-shifts or nights only	16.3%	1.56	[0.99; 2.46]
Papantoniou 2016 rotating night work	9.6%	1.43	[0.79; 2.59]
Total (95% CI)	36.0%	1.20	[0.75; 1.91]
Heterogeneity: Tau^2 = 0.0913; Chi^2 = 4.39, df = 2 (P = 0.11); I^2 = 54%			
chronotype = Intermediate			
Papantoniou 2016 rotating night work	16.3%	1.13	[0.72; 1.78]
**Total (95% CI)**	**100.0%**	**1.18**	**[0.98; 1.42]**

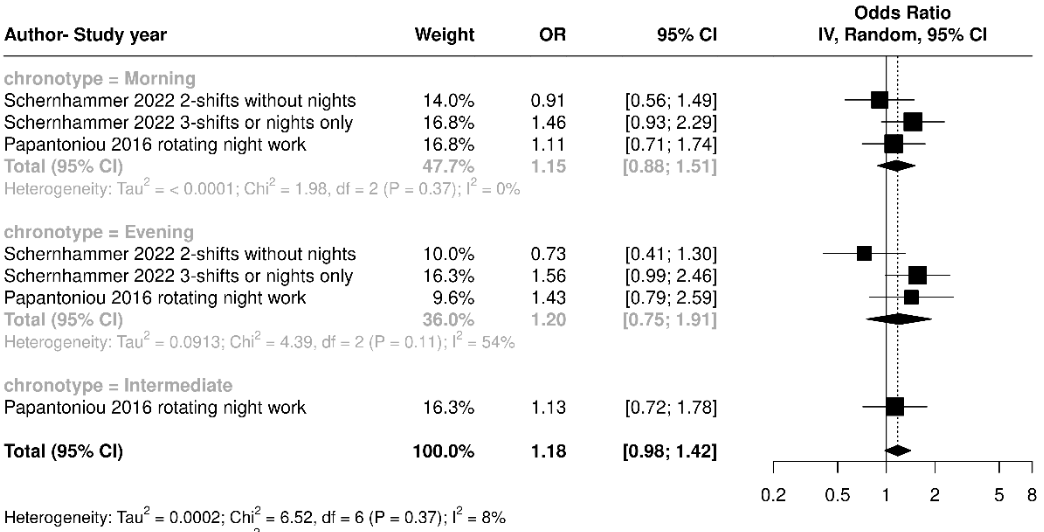

Heterogeneity: Tau^2 = 0.0002; Chi^2 = 6.52, df = 6 (P = 0.37); I^2 = 8%
Test for subgroup differences: Chi^2 = 0.03, df = 2 (P = 0.98)

(b) Prostate cancer

Author- Study year	Weight	OR	95% CI
chronotype = Morning			
Lozano-Lorca 2020 rotating night work	11.8%	1.50	[0.82; 2.75]
Wendeu-Foyet 2018 rotating night work	12.8%	0.43	[0.25; 0.74]
Papantoniou 2014 rotating night work	17.4%	1.12	[0.80; 1.56]
Total (95% CI)	42.0%	0.90	[0.44; 1.84]
Heterogeneity: Tau^2 = 0.3333; Chi^2 = 11.12, df = 2 (P < 0.01); I^2 = 82%			
chronotype = Intermediate			
Lozano-Lorca 2020 rotating night work	6.7%	1.77	[0.66; 4.74]
Wendeu-Foyet 2018 rotating night work	14.6%	1.02	[0.64; 1.62]
Papantoniou 2014 rotating night work	15.5%	1.11	[0.73; 1.69]
Total (95% CI)	36.7%	1.12	[0.83; 1.51]
Heterogeneity: Tau^2 = 0; Chi^2 = 0.99, df = 2 (P = 0.61); I^2 = 0%			
chronotype = Evening			
Lozano-Lorca 2020 rotating night work	3.1%	2.72	[0.54; 13.66]
Wendeu-Foyet 2018 rotating night work	8.2%	1.95	[0.84; 4.53]
Papantoniou 2014 rotating night work	10.0%	1.44	[0.70; 2.95]
Total (95% CI)	21.3%	1.72	[1.03; 2.89]
Heterogeneity: Tau^2 = 0; Chi^2 = 0.63, df = 2 (P = 0.73); I^2 = 0%			
**Total (95% CI)**	**100.0%**	**1.15**	**[0.85; 1.56]**

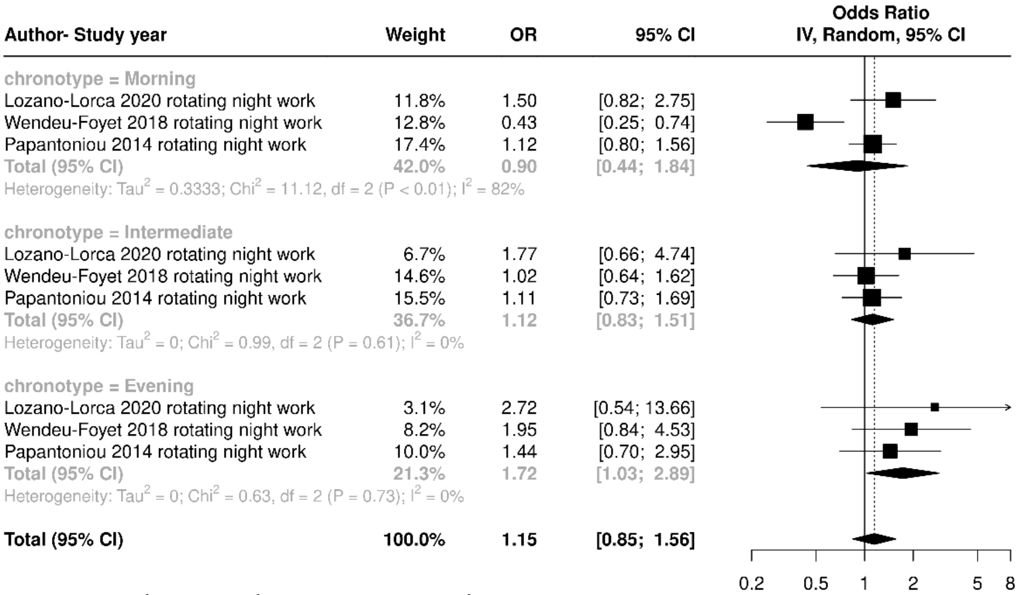

Heterogeneity: Tau^2 = 0.1107; Chi^2 = 16.77, df = 8 (P = 0.03); I^2 = 52%
Test for subgroup differences: Chi^2 = 2.71, df = 2 (P = 0.26)

Supplementary Figure S5 Permanent night shift work by chronotype

(a) Breast cancer

Author- Study year	Weight	OR	95% CI
chronotype = Morning			
Papantoniou 2016 permanent night work	41.5%	1.26	[0.76; 2.09]
chronotype = Intermediate			
Papantoniou 2016 permanent night work	32.6%	1.24	[0.70; 2.19]
chronotype = Evening			
Papantoniou 2016 permanent night work	25.9%	1.11	[0.59; 2.10]
**Total (95% CI)**	**100.0%**	**1.21**	**[0.88; 1.68]**

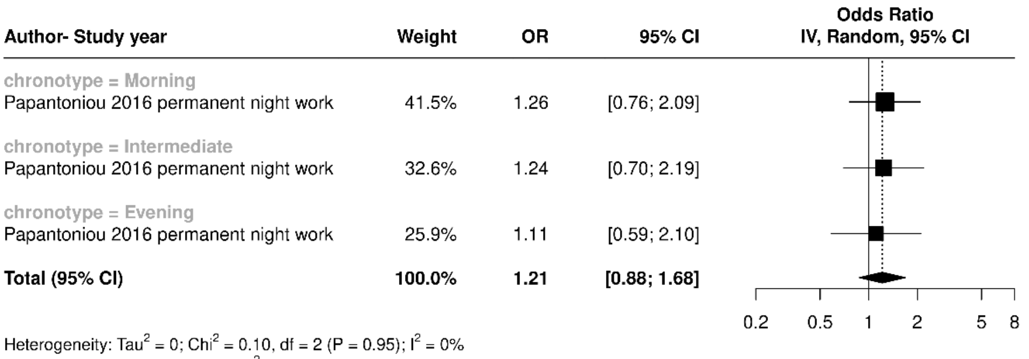

Heterogeneity: $Tau^2 = 0$; $Chi^2 = 0.10$, df = 2 (P = 0.95); $I^2 = 0\%$
Test for subgroup differences: $Chi^2 = 0.10$, df = 2 (P = 0.95)

(b) Prostate cancer

Author- Study year	Weight	OR	95% CI
chronotype = Morning			
Lozano-Lorca 2020 permanent night work	6.4%	0.99	[0.50; 1.95]
Wendeu-Foyet 2018 permanent night work	17.7%	1.02	[0.68; 1.53]
Papantoniou 2014 permanent night work	19.0%	1.19	[0.80; 1.77]
Total (95% CI)	43.2%	1.09	[0.84; 1.41]
Heterogeneity: $Tau^2 = 0$; $Chi^2 = 0.37$, df = 2 (P = 0.83); $I^2 = 0\%$			
chronotype = Intermediate			
Lozano-Lorca 2020 permanent night work	3.0%	1.64	[0.61; 4.40]
Wendeu-Foyet 2018 permanent night work	26.9%	0.93	[0.67; 1.30]
Papantoniou 2014 permanent night work	12.1%	0.90	[0.55; 1.48]
Total (95% CI)	42.0%	0.96	[0.74; 1.25]
Heterogeneity: $Tau^2 = 0$; $Chi^2 = 1.23$, df = 2 (P = 0.54); $I^2 = 0\%$			
chronotype = Evening			
Lozano-Lorca 2020 permanent night work	1.3%	3.53	[0.76; 16.37]
Wendeu-Foyet 2018 permanent night work	8.0%	1.71	[0.93; 3.14]
Papantoniou 2014 permanent night work	5.6%	1.57	[0.76; 3.26]
Total (95% CI)	14.8%	1.76	[1.13; 2.75]
Heterogeneity: $Tau^2 = 0$; $Chi^2 = 0.89$, df = 2 (P = 0.64); $I^2 = 0\%$			
**Total (95% CI)**	**100.0%**	**1.11**	**[0.93; 1.32]**

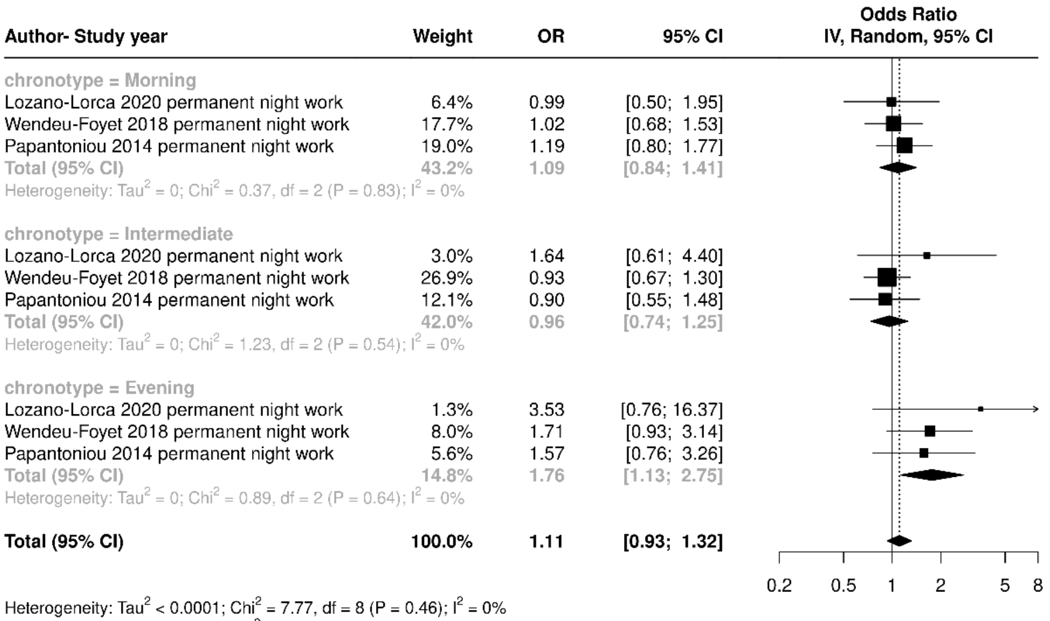

Heterogeneity: $Tau^2 < 0.0001$; $Chi^2 = 7.77$, df = 8 (P = 0.46); $I^2 = 0\%$
Test for subgroup differences: $Chi^2 = 5.28$, df = 2 (P = 0.07)

Supplementary Figure S6 Subgroup analyses by study designs

(a) Breast cancer with case-control study design

Shift work

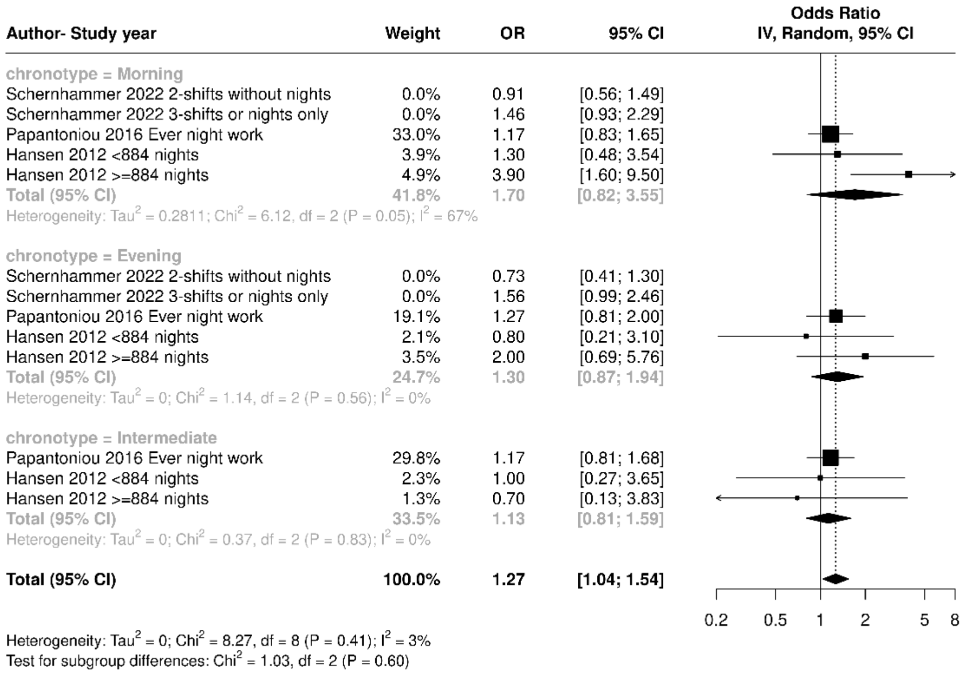

Night shift work

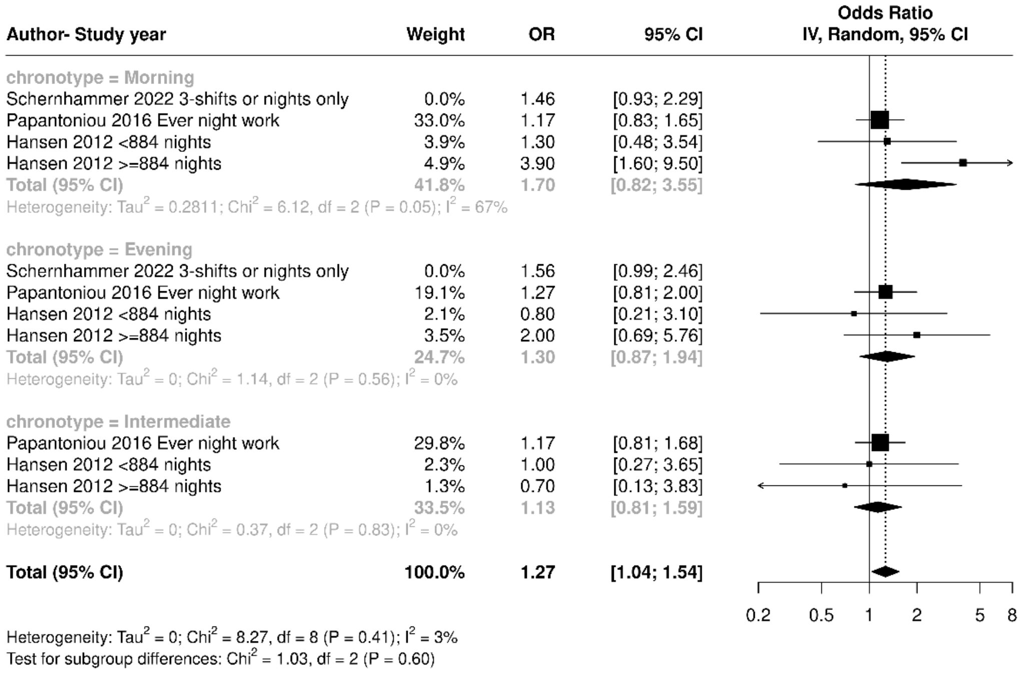

(b) Prostate cancer with case-control study design

Shift work

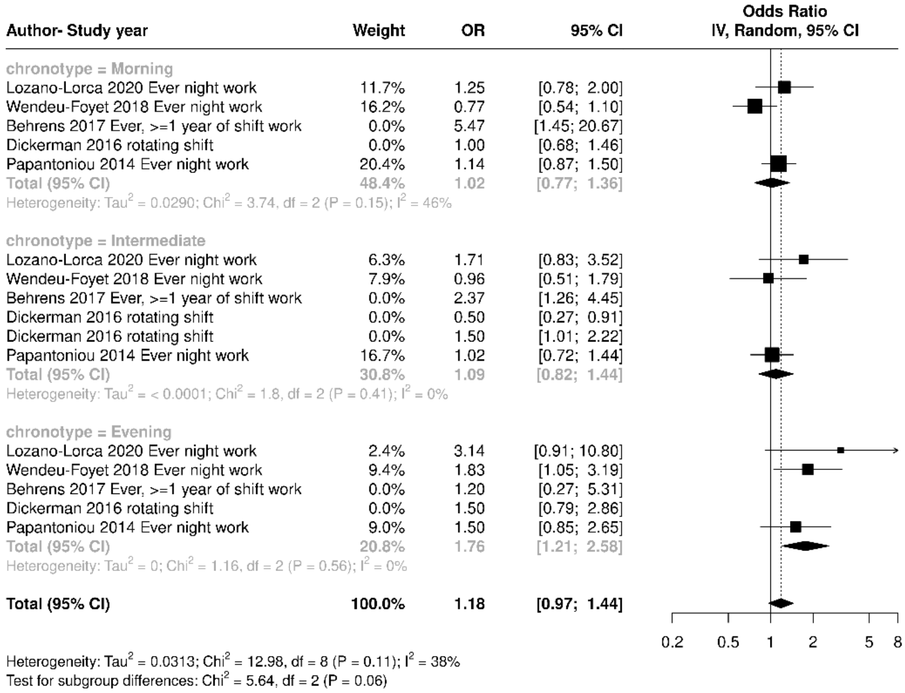

Night shift work

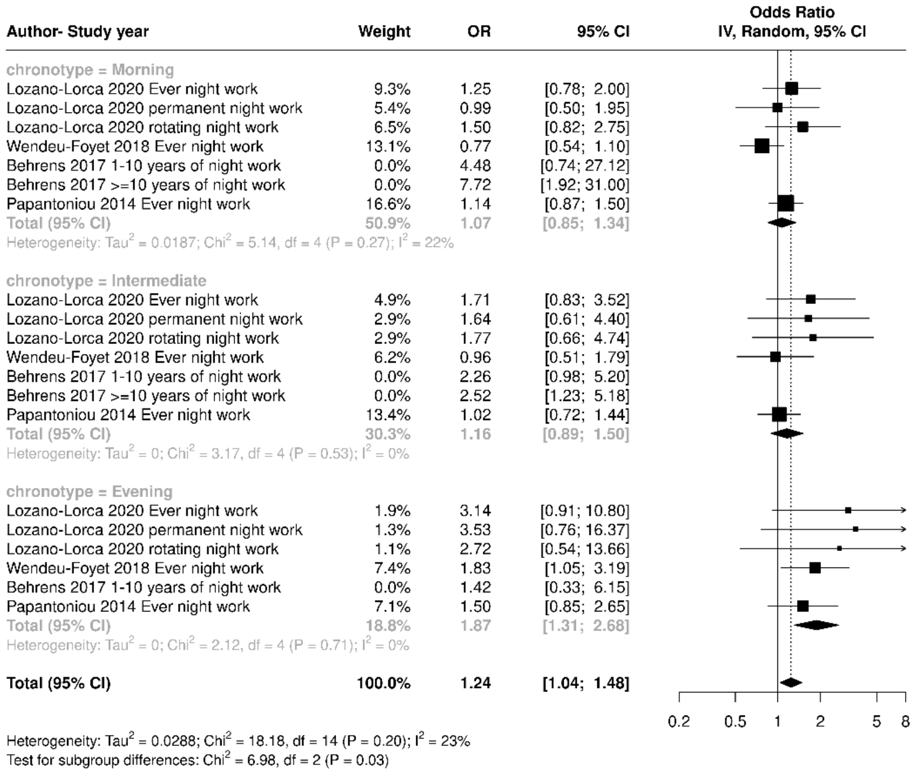

(c) Poor mental health removing the mood disorder study

Shift work

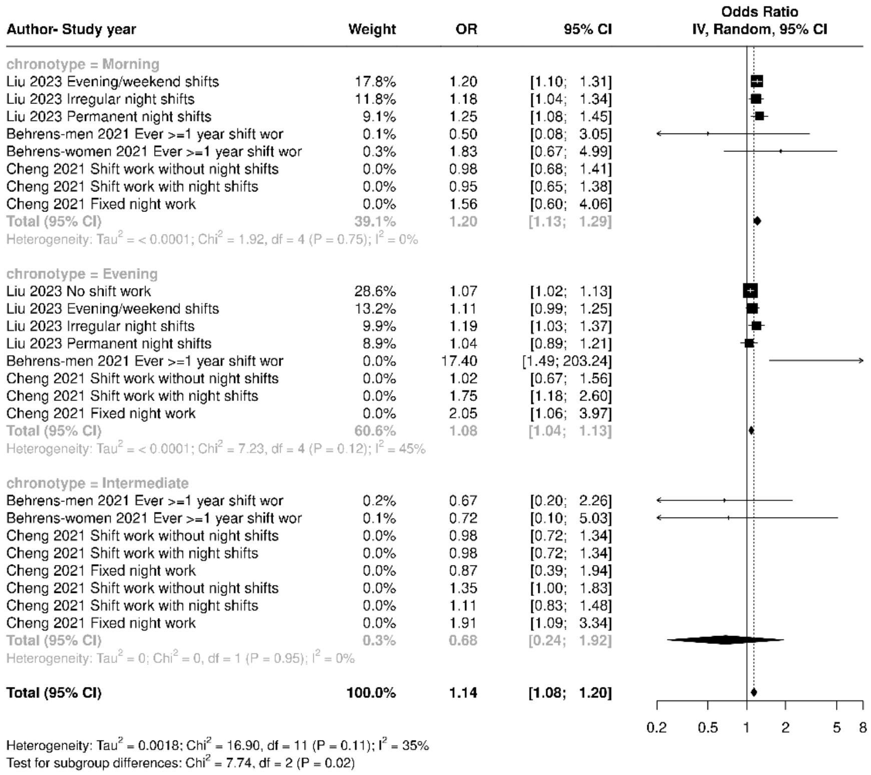

Night shift work

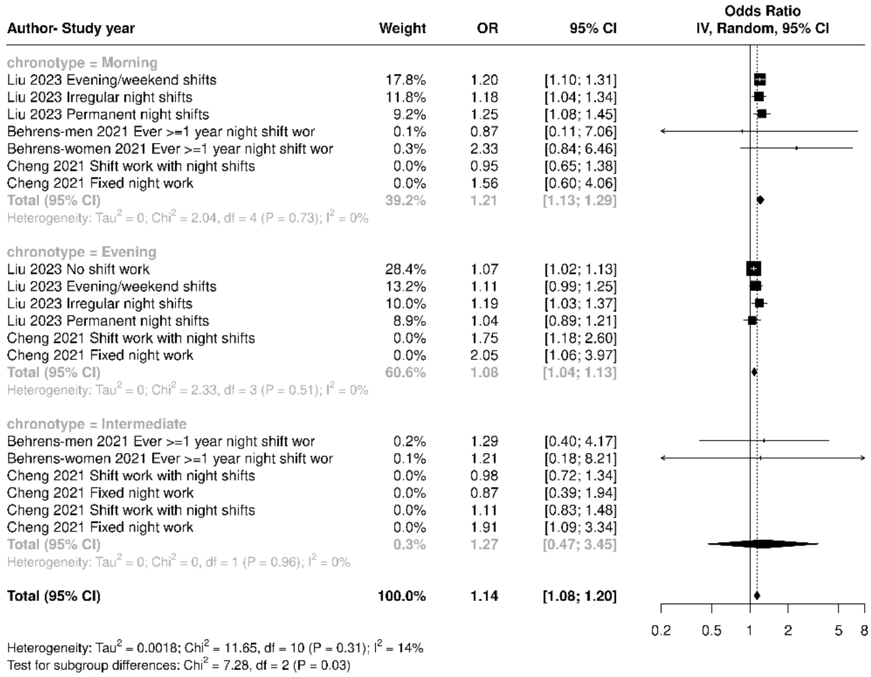

Supplementary Figure S7 Leave-one-out meta-analysis

Study	Tau2	Tau	I2	OR	95% CI
Omitting Schernhammer 2022	0.2620	0.5119	99.0%	1.41	[1.07; 1.86]
Omitting Papantoniou 2016	0.2619	0.5117	99.0%	1.41	[1.07; 1.86]
Omitting Hansen 2012	0.2618	0.5116	99.0%	1.40	[1.06; 1.84]
Omitting Lozano-Lorca 2020	0.2634	0.5132	99.0%	1.39	[1.05; 1.84]
Omitting Wendeu-Foyet 2018	0.2525	0.5025	99.0%	1.44	[1.09; 1.89]
Omitting Behrens 2017	0.2481	0.4981	99.0%	1.35	[1.04; 1.77]
Omitting Dickerman 2016	0.2539	0.5039	99.0%	1.43	[1.09; 1.88]
Omitting Papantoniou 2014	0.2608	0.5107	99.0%	1.42	[1.07; 1.87]
Omitting Hulsegge 2018	0.2577	0.5076	99.0%	1.38	[1.05; 1.82]
Omitting Vetter 2018	0.2609	0.5108	99.0%	1.42	[1.07; 1.87]
Omitting Vetter 2015	0.2632	0.5130	99.0%	1.40	[1.06; 1.85]
Omitting Liu 2023	0.2606	0.5105	98.9%	1.42	[1.08; 1.87]
Omitting Behrens-men 2021	0.2514	0.5014	99.0%	1.42	[1.08; 1.85]
Omitting Behrens-women 2021	0.2547	0.5047	99.0%	1.41	[1.07; 1.84]
Omitting Cheng 2021	0.2608	0.5107	99.0%	1.42	[1.07; 1.87]
Omitting Vetter 2018	< 0.0001	0.0002	41.2%	1.14	[1.11; 1.18]
**Total (95% CI)**	**0.2454**	**0.4954**	**99.0%**	**1.40**	**[1.08; 1.81]**

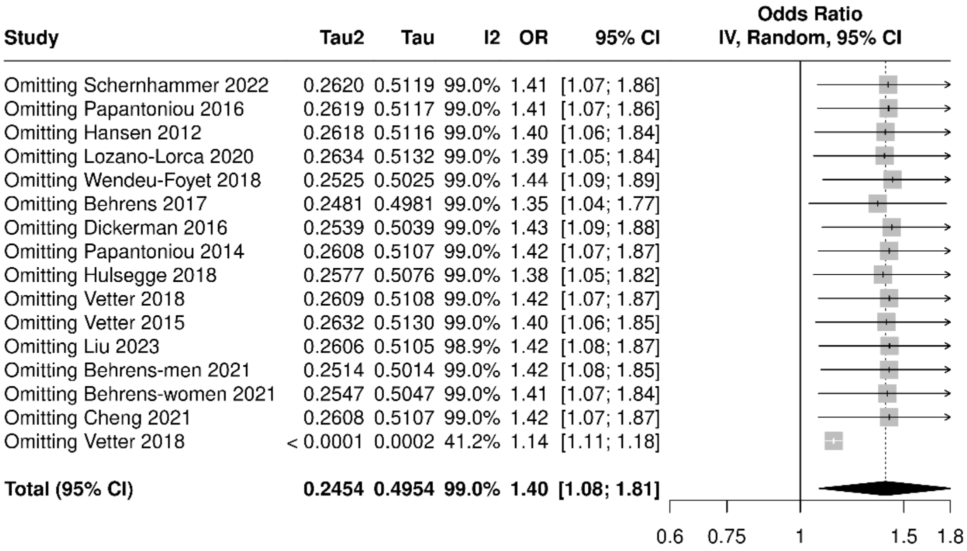

Supplementary Figure S8 Funnel plots

(a) Prostate cancer

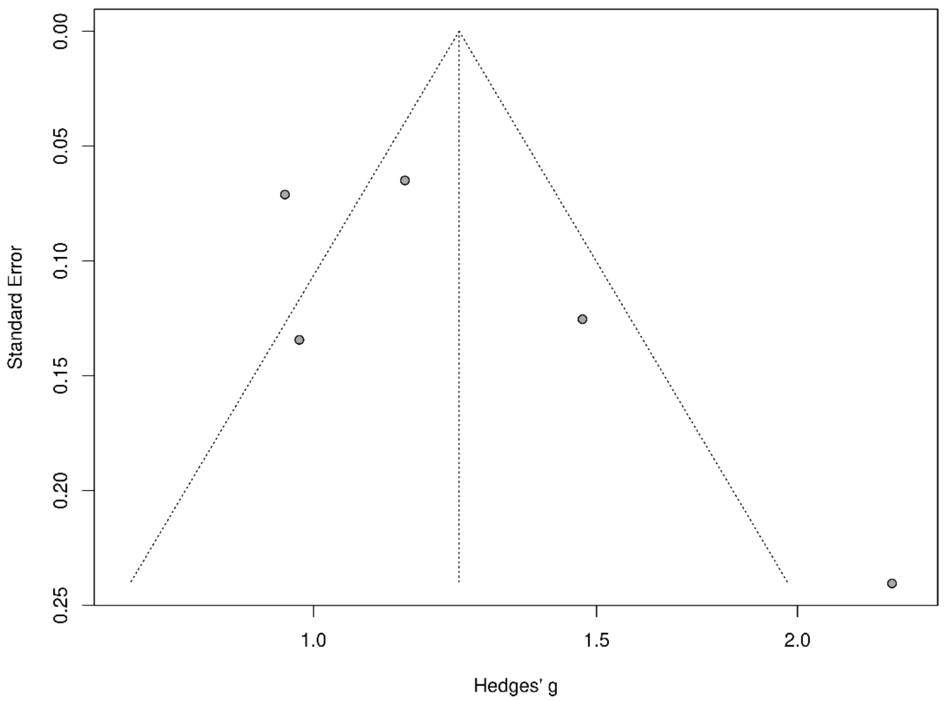

(b) Diabetes

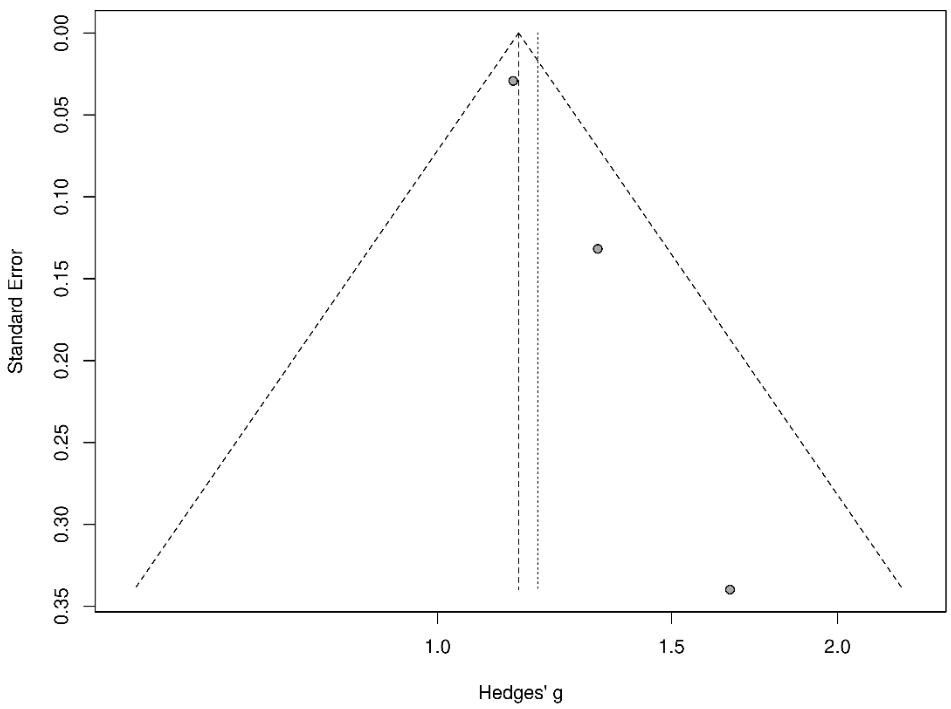

(c) Poor mental health

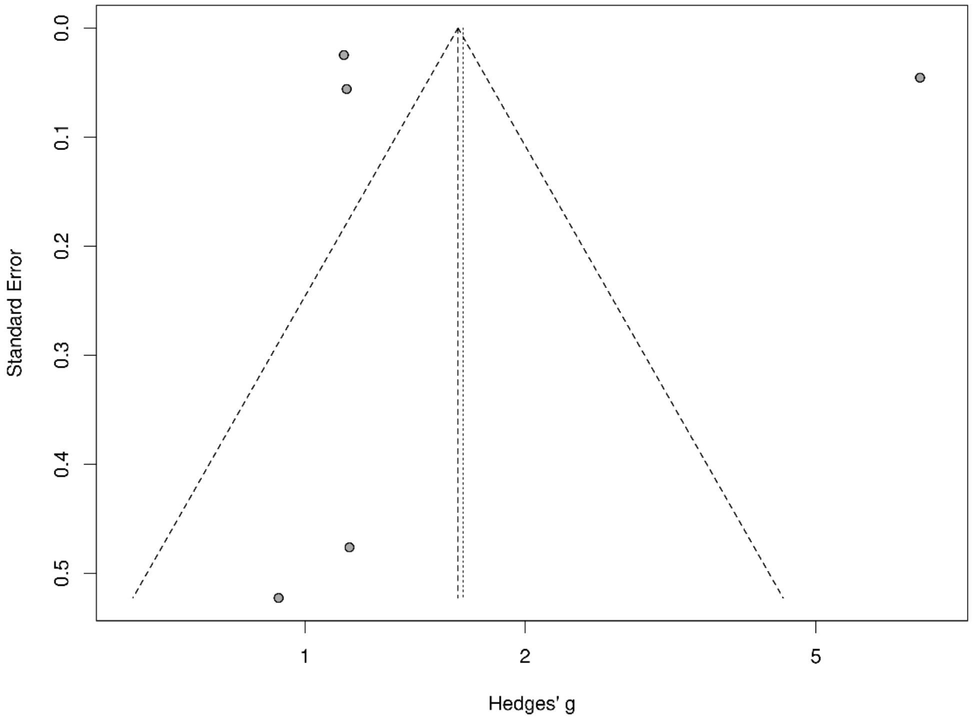

Supplement: Supplementary material [file SJWEH-52-98-S001.pdf]
